# A Neuroanatomically Grounded Optimal Control Model of the Compensatory Eye Movement System in Mice

**DOI:** 10.3389/fnsys.2020.00013

**Published:** 2020-03-25

**Authors:** Peter J. Holland, Tafadzwa M. Sibindi, Marik Ginzburg, Suman Das, Kiki Arkesteijn, Maarten A. Frens, Opher Donchin

**Affiliations:** ^1^Department of Neuroscience, Erasmus MC, Rotterdam, Netherlands; ^2^Department of Biomedical Engineering, Zlotowski Centre for Neuroscience, Ben Gurion University, Beer-Sheva, Israel; ^3^School of Psychology, University of Birmingham, Birmingham, United Kingdom; ^4^Singapore Institute for Neurotechnology, Singapore, Singapore; ^5^Department of Experimental and Applied Psychology, Vrije Universiteit Amsterdam, Amsterdam, Netherlands; ^6^Department of Human Movement Sciences, Vrije Universiteit Amsterdam, Amsterdam, Netherlands; ^7^ABC Centre for Robotics, Ben Gurion University, Beer-Sheva, Israel

**Keywords:** VOR, OKR, mouse, forward model, state estimation, adaptation

## Abstract

We present a working model of the compensatory eye movement system in mice. We challenge the model with a data set of eye movements in mice (*n* =34) recorded in 4 different sinusoidal stimulus conditions with 36 different combinations of frequency (0.1–3.2 Hz) and amplitude (0.5–8°) in each condition. The conditions included vestibular stimulation in the dark (vestibular-ocular reflex, VOR), optokinetic stimulation (optokinetic reflex, OKR), and two combined visual/vestibular conditions (the visual-vestibular ocular reflex, vVOR, and visual suppression of the VOR, sVOR). The model successfully reproduced the eye movements in all conditions, except for minor failures to predict phase when gain was very low. Most importantly, it could explain the interaction of VOR and OKR when the two reflexes are activated simultaneously during vVOR stimulation. In addition to our own data, we also reproduced the behavior of the compensatory eye movement system found in the existing literature. These include its response to sum-of-sines stimuli, its response after lesions of the nucleus prepositus hypoglossi or the flocculus, characteristics of VOR adaptation, and characteristics of drift in the dark. Our model is based on ideas of state prediction and forward modeling that have been widely used in the study of motor control. However, it represents one of the first quantitative efforts to simulate the full range of behaviors of a specific system. The model has two separate processing loops, one for vestibular stimulation and one for visual stimulation. Importantly, state prediction in the visual processing loop depends on a forward model of residual retinal slip after vestibular processing. In addition, we hypothesize that adaptation in the system is primarily adaptation of this model. In other words, VOR adaptation happens primarily in the OKR loop.

## Introduction

Compensatory eye movement (CEM) is a general term for several reflexes whose goal is to maintain a stable image on the retina during movements of the head by moving the eyes in the opposite direction (Delgado-García, 2000). In other words, these reflexes serve to reduce retinal slip (movement of the visual image across the retina). In afoveate animals like mice, the CEM comprises two reflexes: the vestibulo-ocular reflex (VOR) uses vestibular input to predictively compensate retinal slip and the optokinetic reflex (OKR) is driven by the retinal slip itself. The two reflexes have roughly complementary properties: the OKR performs well in low velocities and the VOR works well at high frequencies. The existence of these reflexes allows accurate compensation of retinal slip velocities experienced in normal behavior. However, a challenge for any model of the CEM is to explain the interaction between VOR and OKR. In many conditions, the combined action, with a gain of almost exactly one, is much less than the sum of the two reflexes driven separately. The main aim of this paper is to produce a model of the system and that can simulate the behavior of the VOR, OKR and their interaction. Importantly, the model should be able to reproduce these behaviors with a single set of parameters and be based on the known neuroanatomy. The model must also function in the presence of motor and sensory noise as well as including realistic delays involved in visual sensation.

The CEM system has a number of properties that make it a popular candidate for quantitative modeling of sensorimotor processes (for review see Glasauer, 2007). First, its goal, minimizing retinal slip, is clear and invariant over time (Robinson, 1981). Second, the dynamics of the system as a whole are close to linear. Third, the output only has three degrees of freedom. Moreover, horizontal CEM can be isolated from the other two degrees of freedom and treated as a system with a single degree of freedom. There is a rich tradition in developing models of the CEM (Raphan et al., 1979; Robinson, 1981; Kawato and Gomi, 1992; Merfeld and Young, 1995; Laurens and Droulez, 2007; Lisberger, 2009; Karmali and Merfeld, 2012; Clopath et al., 2014; Laurens and Angelaki, 2017). The different models address different aspects of the CEM system, and we discuss here two key features shared by some of the models: containing an internal model that is used to predict sensory feedback and explaining the interactions of VOR and OKR.

One of the most enduring models is based on the Merfled Observer Model (Merfeld and Young, 1995; Karmali and Merfeld, 2012). In this model the brain uses forward modeling and sensory prediction error to appropriately compensate for unexpected perturbations. However, they only model VOR leaving open the question of the interaction of VOR and OKR. Laurens and Angelaki (2017) similarly propose a model based on internal models and sensory prediction. Their focus is on comparing active and passive movements, but, like Merfeld, they do not concentrate on the interaction of OKR and VOR and they do not consider the actual motor signals that reach the eye and the way that feedback from the eyes influences eye movements. Our model is in the spirit of these two earlier models in that it is based on the hypothesis that the brain uses internal models to predict upcoming sensory signals and make appropriate corrections. However, we are not primarily concerned with estimate of the movements of the body, but rather with reduction of retinal slip. This means we must consider both the VOR and the OKR, their interactions, and the way the system is driven by visual input.

The only models that attempt to combine VOR and OKR, to our knowledge, are Raphan et al. (1979) and the related model of Laurens and Angelaki (2017). While our model has some strong similarities to Raphan et al. (1979), their approach lacks the explicit notion of internal modeling which characterized more recent approaches. Our own model, while similar in important ways to other approaches, is originally based in efforts to connect the CEM with the ideas borrowed from optimal control theory that have been productive in the study of reaching movements (Shadmehr and Krakauer, 2008; Frens and Donchin, 2009; Haar and Donchin, 2019). Optimal control suggests that the motor system operates in a “full feedback” mode: generating motor commands in response to the best guess regarding the current situation as opposed to using a pre-defined plan (Todorov and Jordan, 2002). However, it has proved very difficult to build optimal control models that make specific predictions for real, physiological motor circuits. Our original thinking was that the CEM was a sufficiently simple motor system to allow for the use of this framework. In the end, our model is consistent with earlier models in the field but extends them by combining VOR and OKR and internal modeling in a manner consistent with the optimal feedback approach used in other motor fields.

**Figure 1:**
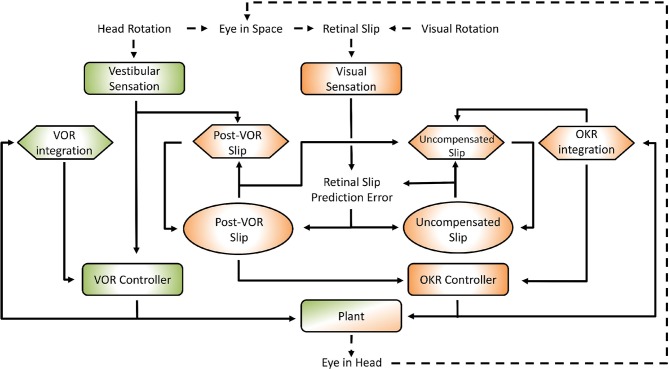
General layout of the model. Green areas are vestibular, orange areas are optokinetic. Hexagons represent Forward Models, ellipses are State Estimators. Dashed arrows indicate processes in the real world, solid arrows are neural processes. Details of the model are specified in the text and Supplementary Material. The connection between the vestibular sensation and the Post-VOR Slip forward model is one of the main innovations in the model and represents the OKR modeling the inaccuracies in the VOR loop.

We build a working quantitative model (Figure 1) of the compensatory eye movement system (CEM) starting from the ideas developed in the Frens and Donchin state predicting feedback control (SPFC) scheme (Frens and Donchin, 2009). It explains data collected from CEM in mice across a broad range of frequencies and amplitudes and different stimulation conditions. The model reproduces the main characteristics of mouse vVOR (rotation of the animal in the light, providing simultaneous visual and vestibular stimulation). Importantly, the same set of parameters also results in good predictions of responses in VOR, OKR and additional conditions, i.e., suppressed VOR (sVOR; simultaneous rotation of the animal and its visual surroundings), and responses to sum-of-sines (SOS) stimuli. To test a hypothetical mapping of the model onto the underlying anatomy, we simulate lesions in specific parts of the model and compare the results with actual lesion studies in mice. Finally, the model also successfully captures VOR adaptation. We introduce the novel proposal that VOR adaptation actually occurs through changes in the way OKR predicts inaccuracies in the VOR.

The interaction of the VOR and OKR might be explained if OKR is capable of predicting the retinal slip not compensated by VOR. This is at the core of the model that we present. The current model is essentially hierarchical, with the vestibular and the visual components of the CEM handled in two distinct loops (see Figure 1). This is close to the traditional view of CEM which also incorporates two, more or less separate, mechanisms for the VOR and OKR (Wakita et al., 2017). The VOR operates in a partially open-loop fashion with feedback used to drive only the forward model of the eye without modifying processing of the vestibular state itself. In our model the OKR loop, on the other hand, incorporates forward models of the eye, the visual input, and also the VOR system. That is, the OKR not only predicts current retinal slip based on models of the environment and the eye movements, it also incorporates a model of the residual retinal slip that remains after the actions of the VOR loop. This represents a crucial expansion of previous models in which sensory systems and plant dynamics have been included in the internal model, so that now the VOR reflex itself is modeled by the OKR system (Post-VOR Slip, Figure 1). The sensitivity of the OKR loop's estimate of inaccuracies in the VOR loop is determined by a single parameter, ζ (see Equation 5, Equation 29 in Supplementary Material). An additional test of our model is that it should be possible to set the value of ζ adaptively, thus mimicking VOR adaptation. Thus, adaptation of the CEM system (at least to first approximation) is mostly adaptation of the OKR model of VOR inaccuracies (Figure 1; Post-VOR Slip). This is consistent with experimental findings (as reviewed in the discussion) and also with our hypothesis that the OKR loop is more dependent on forward model prediction than the VOR.

We chose to model and perform experiments in mice because mice, being afoveate, lack a confounding smooth pursuit system. Moreover, we concern ourselves only with the horizontal CEM as is commonly done in the experimental literature. However, since rotations are non-commutative, expanding the model to three dimensions is not trivial and this is an area that has been tackled in other models (Merfeld and Young, 1995; Laurens and Angelaki, 2017). We ultimately decided to constrain ourselves to modeling only the horizontal CEM as there exists a great deal more literature on this than the vertical or torsional responses in mice. In order to compare our model to data, we collected from mice in a large set of conditions (VOR, OKR, vVOR, sVOR, SOS), frequencies and amplitudes. Such a data set was lacking in the literature so that our contribution in this work, beyond a model that fits all existing data, is a comprehensive data set showing CEM behavior across a complete array of stimuli. Importantly, the Matlab code for implementing the model as well as the behavioral data and analysis code are all freely available online to encourage the extension of the model into untested conditions.

## Materials and Methods

### Model

The model was implemented in Matlab (version 2016a; The MathWorks, Natick, MA, USA) and calculations were performed via matrix multiplication with a time step of 1 ms. In describing the model we first provide a brief outline of the neuroanatomical basis and subsequently outline our approach to modeling. We do this separately for the VOR and OKR parts of the model (green and orange areas in Figure 1, respectively). In the Model Specification section we provide a summary of the mathematical specification of the model, with full details provided in the Supplementary Material.

#### VOR

The mouse VOR uses vestibular input from the semi-circular canals (labyrinth) to compensate head movement (Delgado-García, 2000). Vestibular afferents from the labyrinth project directly to VN with a small delay (2 ms; Sohmer et al., 1999). Their activity accurately reflects head velocity at high frequencies but not at low frequencies (Robinson, 1981) due to filtering properties of the vestibular labyrinth (Yang and Hullar, 2007).

Thus, in modeling VOR, the processing is quite simple (green areas in Figure 1). Since the system has no access to the actual head velocity, we use the vestibular signal as an approximation of the head velocity. Neither system dynamics nor the oculomotor command affect head dynamics. Note, therefore, that this model currently does not distinguish between active and passive head movements, i.e., it does not incorporate efference copy or proprioceptive information about head movement.

#### OKR

In the mouse, the OKR originates in velocity sensitive neurons of the retina, which project through the Accessory Optic System (AOS) and Nucleus Reticularis Tegmenti Pontis (NRTP) to the vestibular nucleus (VN) and the vestibulo-cerebellum (Gerrits et al., 1984; Langer et al., 1985; Glickstein et al., 1994). The VN output is sent to the brainstem nuclei, which drive the extra-ocular muscles. In the case of horizontal eye movements, these are the abducens nucleus (Ab), the oculomotor nucleus (OMN), and nucleus prepositus hypoglossi (NPH; Büttner-Ennever and Büttner, 1992). The OKR has a species-dependent response delay of 70–120 ms (Collewijn, 1969; van Alphen et al., 2001; Winkelman and Frens, 2006) primarily caused by the visual processing in the pathway from retina to VN (Graf et al., 1988). The retinal afferents saturate at high velocities (Oyster et al., 1972; Soodak and Simpson, 1988), causing non-linearities in the OKR in this range (Collewijn, 1969; van Alphen et al., 2001). Thus, the OKR is ineffective in compensating high velocity (and thus often high frequency) visual stimuli.

One main innovation in our model is that the OKR system assumes that the VOR only compensates for some proportion of the head movement. The role of the rest of the control system (orange areas in Figure 1) is to estimate the retinal slip that will remain after the action of the VOR loop (Post-VOR Slip) and provide this information for the OKR controller. Post-VOR slip arises from two sources: from changes in the velocity of the visual stimulus and from head movements not compensated by the VOR. Thus, our forward model estimate of movement of the visual surrounding (Post-VOR Slip; left orange hexagon in Figure 1) will be updated by a factor proportional to head acceleration (Equation 5, See also Equation 29 in Supplementary Material). The specific constant of proportionality, ζ, is discussed in the section on VOR adaptation below. The combination of this predicted retinal slip (Post-VOR Slip) combined with an estimate of how much the OKR is moving the eye, gives the OKR's forward model prediction of uncompensated retinal slip (right orange hexagon in Figure 1).

As one can see in Figure 1, state estimation produces estimates of both Post-VOR slip, and uncompensated retinal slip (oval boxes). Post-VOR slip is retinal slip after VOR compensation and uncompensated slip is that remaining after the action of both systems. In both cases, we chose to use an approximation of a Kalman filter to perform state estimation. Kalman filters estimate state using an optimal combination of previous state and incoming sensory data, optimized relative to the variance associated with each of them (Porrill et al., 2013). That is, they generate the estimate which is most likely to be closest to the true value, given their inputs. In our case, the two inputs were not optimally mixed, but rather the mixing was chosen to match the data (see Supplementary Material, Equation 42). We are not claiming that the mouse brain implements a true optimal Kalman filter, but rather some weighted mixing of sensory input and forward model prediction. Thus, through the model architecture, vestibular input only affects our estimate of the head velocity, and retinal input affects both our estimate of retinal slip and our estimate of uncompensated retinal slip.

#### VOR Adaptation

VOR adaptation occurs when gaze consistently fails to compensate head movement (Blazquez et al., 2004; Schonewille et al., 2010; Shin et al., 2014). In a laboratory environment, a rotating visual environment can lead such failure (as described in the Methods below). This causes persistent changes in the VOR, such that retinal slip is reduced in the new situation. In our model, such a mismatch would affect the proportionality constant ζ. This is because the OKR system's assumption that retinal slip is the result of inaccuracies in the VOR loop.

#### Summary of Model Specification

What follows is a brief description of the mathematical specification of the model. A full description is available in the Supplementary Material. We use a standard linear systems formulation (Frens and Donchin, 2009):

(1) $$\begin{array}{l} x_{k+1}=Ax_{k}+Bu_{k}+z_{k}+n_{k}\\ \;y_{k}=Dx_{k} \end{array}$$

with dynamics *A* applied to system state, *x*_*k*_, which is also affected by the command signal, *u*_*k*_, the external state of the world, *z*_*k*_, and noise, *n*_*k*_. Finally, this state leads to sensory input, *y*_*k*_. *z*_*k*_ is the external input and includes the change in the actual head velocity, vestibular sensory signal, and movement of the visual stimulus.

The state vector includes some easily recognizable quantities like head state (position, *H*, and velocity, $\dot{H}$), eye state (*E* and $\dot{T}$) and state of the visual scene (*T* and $\dot{T}$). However, it also includes two enigmatic variables (*V* and *R*). One is the filtered head velocity signal that represents vestibular input. The semicircular canals are described as a high pass filter that acts on head velocity:

(2) $$\begin{array}{l} \overset{∙}{V}=-\frac{1}{T_{v}}V+\dot{H} \end{array}$$

Where *V* is the neural signal generated by the velocity sensitive vestibular afferents (as can be seen by comparing with the Supplementary Material, the equations here are simplified for clarity). *T*_*V*_ is the time constant of the filter.

The other more abstract quantity is the combination of head, eye, and visual input that creates retinal slip (*R* = $\dot{H}$ + $\dot{T}$ − $\dot{T}$ with a saturation cutoff at *R*_max_ = 0.65deg/sec). Input delays are represented by including time delayed versions of retinal slip and vestibular input in the state vector and only the delayed versions are available to the internal state estimation. Thus, the state vector is:

(3) $$\begin{array}{l} x=\left[\begin{array}{ccccccccc} H & \dot{H} & V & E & Ė & T & \dot{T} & R & \cdots \end{array}\right] \end{array}$$

with the three dots indicating extra time-delayed copies of vestibular and retinal signals.

In modeling the noise, we opted for model simplicity over realistic modeling of the noise. We followed Todorov (2004) and Harris and Wolpert (1998) in making noise magnitude proportional to the signal. We ran the model with different constants of proportionality for the noise and did not see a change in the results. Given that we have no available data on the amount of sensory or motor noise in the system we used values well in the middle of stable range.

In additional to the external state and the input, we also modeled the controller itself. Our controller is split into two parts, one for the VOR (subscripted *V*) and one for the OKR (subscripted *R*):

(4) $$\begin{array}{l} \begin{array}{cc} \begin{array}{c} {\hat{x}}_{\text{V},k+1}={A^{\prime }}_{\text{V}}{\tilde{x}}_{\text{V},k}+B_{\text{V}}u_{\text{V},k}\\ {\tilde{x}}_{\text{V},k+1}={\hat{x}}_{\text{V},k+1}\\ \;+K_{\text{V}}\left({\overset{∙}{V}}_{k-\delta_{V}}-{D^{\prime }}_{\text{V}}{\hat{x}}_{V,k+1}\right) \end{array} & \begin{array}{c} {\hat{x}}_{\text{R},k+1}=A_{\text{R}}^{\prime }{\tilde{x}}_{\text{R},k}+B_{\text{R}}u_{\text{R},k}\\ {\tilde{x}}_{\text{R},k+1}={\hat{x}}_{\text{R},k+1}\\ \;+K_{\text{R}}\left(R_{k-\delta_{R}}-h\left({\tilde{R}}_{k}\right)\right) \end{array} \end{array} \end{array}$$

Both parts of the controller have the same structure: a forward model of the dynamics (first equation in each) followed by state estimation, which combines forward model prediction with sensory input (second equation in each). Note that sensory inputs, ${\overset{∙}{V}}_{k-\delta_{V}}$ and *R*_*k*−_δ__*R*__, are the delayed versions and that internal estimates of retinal slip must be saturated [*h*(•)] to allow meaningful comparison to sensed retinal slip. *K*_*V*_ and *K*_*R*_ are mixing constants that determine the relative weight of forward model output and sensory input. The vestibular system weights sensory input very heavily while the retinal system weights prediction more heavily.

We use hat notation, $\hat{x}$, for estimates produced by the forward model and tilde notation, $\tilde{x}$, for the combined state estimate. The internal state of the controller, represented by $\hat{x}$ and $\tilde{x}$, represents internal estimates of the system state (Equation 3) described above with some additions. First, it contains two different estimates of eye state, separately represented by the VOR and OKR controllers. This allows the controller to make different calculations. Second, it contains two different estimates of retinal slip. The first is called the uncompensated retinal slip. It reflects an estimate of the retinal slip that remains after both the VOR and OKR contributions. This is the $\tilde{R}$ that appears in the equation above (with the tilde indicating that it is a state estimate resulting from a Kalman filter calculation and forward modeling). It is compared to the actual retinal slip to produce retinal slip prediction error.

The other estimate of retinal slip in the internal state is called the Post-VOR slip, ${\tilde{R}}^{*}$. It reflects an estimate of the amount of retinal slip that will remain after the VOR contribution. It is used to determine the OKR controller output. This estimate exists because the OKR controller assumes that that some head movement remains uncompensated by the VOR. The forward model estimate of uncompensated post-VOR retinal is thus updated by a factor proportional to head acceleration:

(5) $$\begin{array}{l} {\hat{R}}_{k+1}^{*}={\tilde{R}}_{k}^{*}+\zeta \left({\hat{\dot{H}}}_{k}-{\hat{\dot{H}}}_{k-1}\right) \end{array}$$

The constant of proportionality, ζ, is the quantity that is actually being estimated by VOR adaptation. Our data was best fit by using ζ = −0.6 which means that OKR assumes VOR tends to overcompensate for head rotation.

Finally, the motor command for both OKR and VOR is generated by using the equations: $u_{R}=-L_{R}\cdot {\tilde{x}}_{R}$ and $u_{V}=-L_{V}\cdot {\tilde{x}}_{V}$ and *u* = *u*_*R*_ + *u*_*V*_ where *L*_*R*_ and *L*_*V*_ are called the command policy. These are linear functions of the internal estimate of state and the command policy was calculated using an approximation of the equations in optimal control theory (The Riccati equations for a linear-quadratic-regulator, please see Supplementary Material for more details). Other approaches to finding a reasonable control policy are also possible (Harris and Waddington, 2013). The controller must, in any case, compensate for the estimated error while correcting for known dynamics of the motor plant and different ways of reaching similar solutions exist.

#### Parameters

In the model only a few parameters were set to match the data. They were set to match data in the vVOR condition and then the same parameters were used for all conditions. Most variables were either taken from literature, or experimentally derived by us in separate experiments. Interestingly, it turned out that the model produced very similar behavior across a wide range of values for most parameters although it was sensitive to a few parameters (see Table 1). As much as possible, parameters were determined from the literature or from our own data. For example, we determined the maximum VOR and OKR gains from our own data. We used the response to high frequency stimulation to set the maximum gain of the VOR in the model and the response to low velocity stimulation to set the maximum gain of the OKR in the model. The response of the retina to retinal slip saturates at high velocities leading to non-linearity in the response, the value of the parameter representing the saturation point (*R*_max_) was fit to published results (Oyster et al., 1972; Soodak and Simpson, 1988). On the other hand, the filter of the vestibular afferents was shaped to achieve the best fit to the data. Ultimately, the filter that fit our data best was also compatible with the literature. We used a first order high pass filter with a time constant of 4 s (Yang and Hullar, 2007). Similarly, drift velocity and VOR adaptation speed were fit to data and later found to be compatible with the literature (Stahl et al., 2006; Schonewille et al., 2010).

**Table 1:** Overview of all parameters used in the model, their values, the equations they are used (described in Supplementary Material), and a short description of their meaning.

	**Value**	**Equations**	**Meaning**	**Is it critical?**	**How was it set?**
*dt*	1 ms		Time step		
*T*_*p*_	0.5 s	(1, 2, 15, 17, 24, 33, 38)	Leaky integrator time constant for motor nuclei	No	Stahl and Simpson, 1995; Stahl et al., 2015
*T*_*v*_	4 s	(3, 15)	High pass filter constant for the vestibular inputs	yes	Fit to data. Close to value found for actual vestibular afferents by Yang and Hullar (2007) (3 s)
δ_*V*_	2 ms	(5, 20)	Vestibular sensory delay	No	
*a*_*V*_	0.1	(6, 16)	Vestibular sensory noise proportionality constant	No	Middle of the stable range
*R*_max_	0.65 deg/s	(8)	Retinal saturation	yes	Oyster et al., 1972
δ_*R*_	70 ms	(9, 20, 31)	Visual processing delay	yes	van Alphen et al., 2001
*a*_*R*_	0.1	(10, 16)	Visual sensory noise proportionality constant	No	Middle of the stable range
*a*_*u*_	0.1	(15, 16)	Motor noise	No	Middle of the stable range
ζ	−0.6	(29, 33, 38, 48)	Assumed VOR inaccuracy	No	Fit to match VOR performance in the dark.
κ_*V*_	1	(22, 42)	Kalman gain of vestibular input	No	Set so VOR is not eliminated by floccular lesion
κ_*T*_	0.05	(30, 42)	Kalman gain for the effect of retinal slip prediction error on assumed external motion (post-VOR retinal slip)	No	Fit to data
κ_*R, k*_	0.05	(31, 35, 42)	Kalman gain for the effect of retinal slip prediction error on estimate of uncompensated retinal slip	No	Fit to data
γ	$e^{\frac{1}{150}}$	(44)	Discount parameter for cost function	No	Fit to produce credible drift in the dark
θ	2	(44)	Weight of position factor in cost function	No	Fit to produce credible drift in the dark
	100,000		Number of terms kept in infinite cost function sum	No	Arbitrary

*The last two columns describe whether they are critical, and how they were set. We determined how critical the parameters were, by varying them over an order of magnitude, and observing the changes in results*.

### Animals

In order to test the model we recorded CEM in 13 C57Bl/6J mice (Charles River, Wilmington, MA, USA). C57Bl/6J mice are commonly used in oculomotor research enabling comparison of our results to previously published data. We employed four different paradigms i.e., OKR, VOR, sVOR, and vVOR and in each condition we tested a wide range of frequency and amplitude combinations. Details on the experiments are described in the Supplementary Material. Additionally, we measured the drift of the eye back to a central position in the dark (*N* = 6) and the rate of adaptation of the VOR (*N* = 7), full details of the methods are described in the Supplementary Material. All experiments were performed with approval of the local ethics committee and were in accordance with the European Communities Council Directive (86/609/EEC).

Prior to all eye movement recordings, mice underwent surgery to prepare them for head fixation and were allowed sufficient time to recover, details are provided in the Supplementary Material and the full procedure is described in van Alphen et al. (2009).

**Figure 2:**
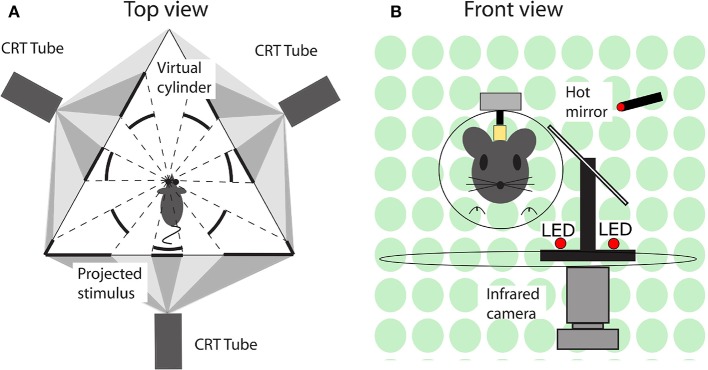
Schematic representation of the experimental setup. **(A)** Top view. A mouse in the setup, with its left eye in the center and surrounded by three screens on which the visual stimuli are projected. The visual stimuli were programmed and displayed in such a way that from the point of view of mouse it appeared as a virtual sphere. **(B)** Front view. A mouse placed in front of a hot mirror, which enabled the infrared camera underneath the table to record the eye movements.

During an experimental session, mice were immobilized by placing them in a plastic tube with the head protruding and the head fixation attached to the turntable with the eye in the central position. Eye movements were recorded via an infra-red video system (Iscan ETL-200, Iscan, Burlington, MA, USA) at a frequency of 120 Hz. Visual stimuli were presented using a modified projector (Christie Digital Systems, Cypress, CA, USA) displaying a panoramic field of 1,592 green dots on virtual sphere fully surrounding the animal. Rotation of the sphere around the vertical axis provided the moving stimuli. Vestibular stimulation was provided via a motorized turntable Mavilor-DC motor 80 (Mavilor Motors S.A., Barcelona, Spain) on which the mouse and eye movement recording system were mounted. Further details are provided in the Supplementary Material and a schematic representation of the stimulus and eye movement recording apparatus in Figure 2.

The VOR adaptation experimental paradigm consisted of an identical stimulus setup with the animal undergoing 6 VOR trials (1 min duration, 1 Hz, 5°) to measure the gain alternating with 5 sVOR trials (5 min duration, 1 Hz, 5°) to induce adaptation.

In the Sum-of-sines (SoS) conditions, the two constituent frequencies were chosen that had no harmonic relation. Four SoS frequency combinations were used in this study: 0.6/0.8, 0.6/1.0, 0.8/1.0, and 1.0/1.9 Hz. Amplitude was either one or two degrees for each frequency component. Either both frequencies had the same amplitude (both 1° or both 2°) or they had different amplitude (one at 1° and the other at 2°). This led to a total of 24 types of stimuli in each of the OKR, VOR, vVOR, and sVOR SoS conditions. Eight mice were used in this paradigm and they all performed all conditions.

### Data Analysis

Every mouse was tested once in each condition, and each stimulus consisted of at least five cycles. Only cycles after the initial transients of the response had decayed were included in analysis. Full details of the analysis details are provided in the Supplementary Material. Briefly, following filtering and removal of fast phase eye movements gain and phase data was calculated by a Bayesian fitting procedure in OpenBugs (Version 3.2.3, http://www.openbugs.net, Lunn et al., 2009) and Matlab curve fitting routines, for single sinusoid stimuli and for SoS stimuli, respectively. The Matlab code and data required for replication of the analysis presented in this paper is available on the Open Science Framework website (https://osf.io/feq7c/).

## Results

### Responses to Sinusoidal Stimulation

**Figure 3:**
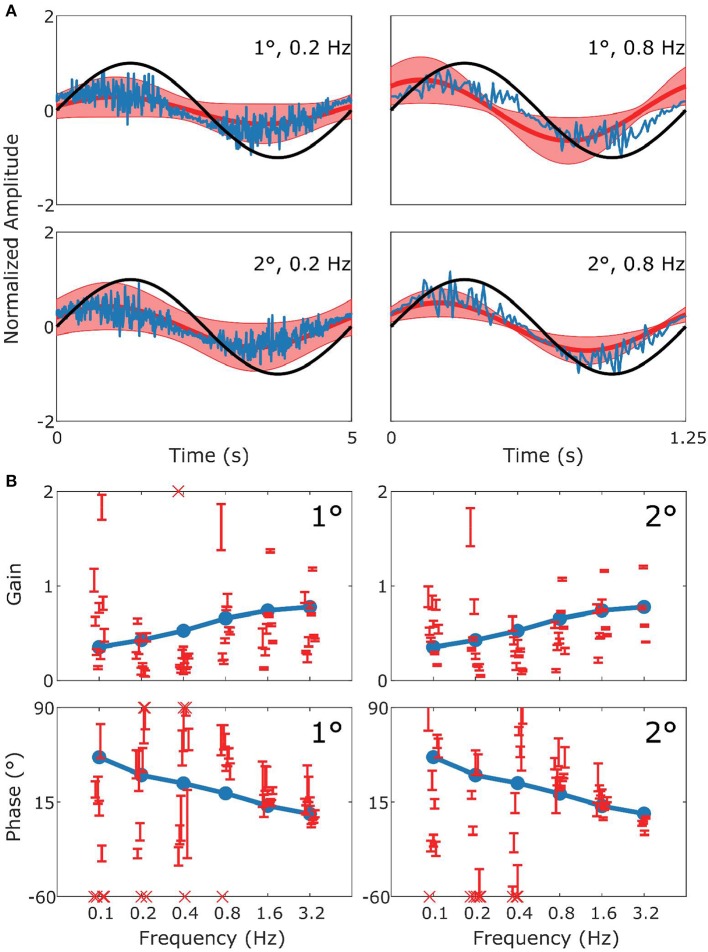
Summary of VOR data and simulation. In **(A)** the upper row displays results for 1° stimuli, the lower row for 2° stimuli. The panels show the stimulus in black (left: 0.2 Hz; right 0.8 Hz), with the simulated response (blue) and the mean measured responses (red). Shaded red regions represent the standard deviation (SD) of the sample of mice showing that the model performance is credible given population variability. **(B)** Are Bode plots for Gain (top panels) and Phase (bottom panels) for the simulated response (blue), individual mice with SD (red error bars). Crosses in the Bode plots indicate data that extend beyond the visible axes. The left and right sides of **(B)** represent bode plots for 1° and 2° stimuli, respectively. Other stimulus conditions fit equally well.

**Figure 4:**
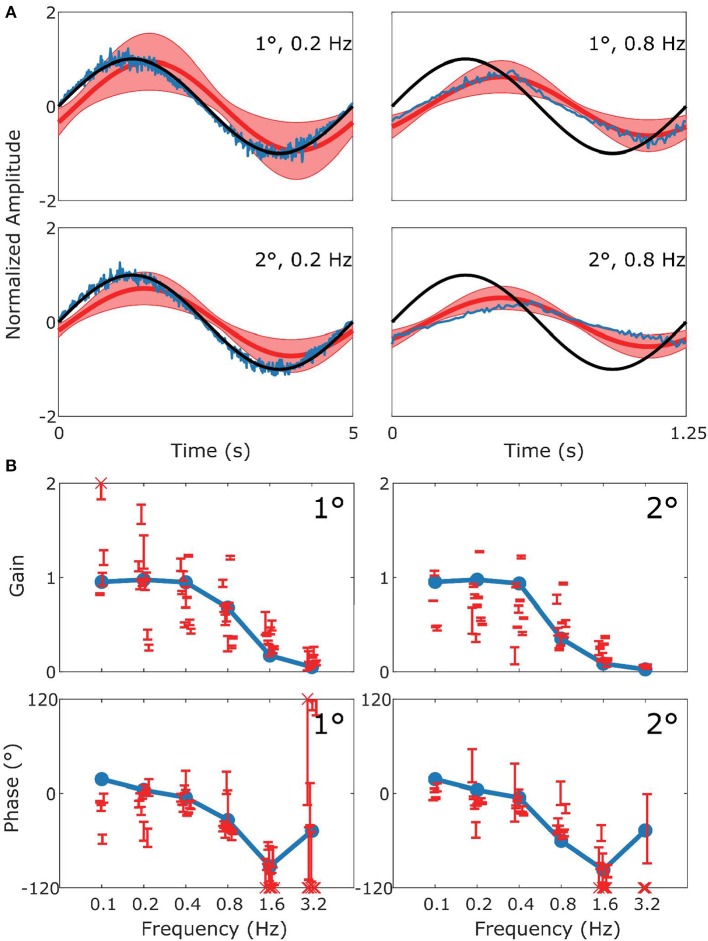
Summary of OKR data and simulation. This figure follows the format of Figure 3, with panel **(A)** displaying the stimulus (black), model response (blue) and measured response (red). **(B)** Are Bode plots for Gain (top panels) and Phase (bottom panels). Note that the phase response of stimuli with Gains < 0.25 could often not reliably be determined.

**Figure 5:**
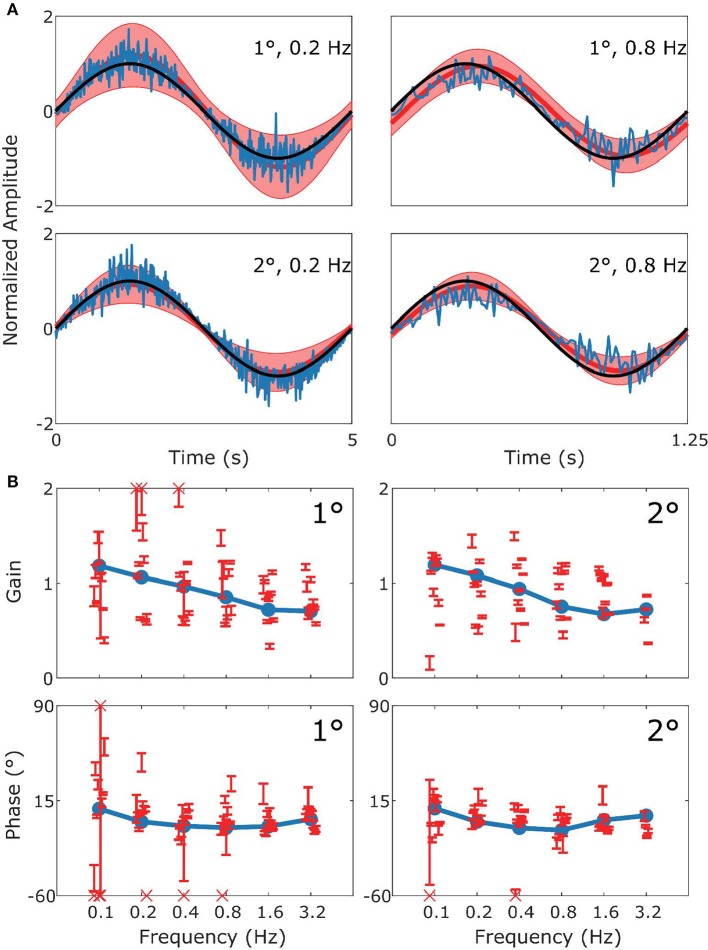
Summary of vVOR data and simulation. This figure follows the format of Figure 3, with panel **(A)** displaying the stimulus (black), model response (blue) and measured response (red). **(B)** Are Bode plots for Gain (top panels) and Phase (bottom panels). Across the whole frequency range tested in both amplitudes there was a very good match of model to experimental data.

**Figure 6:**
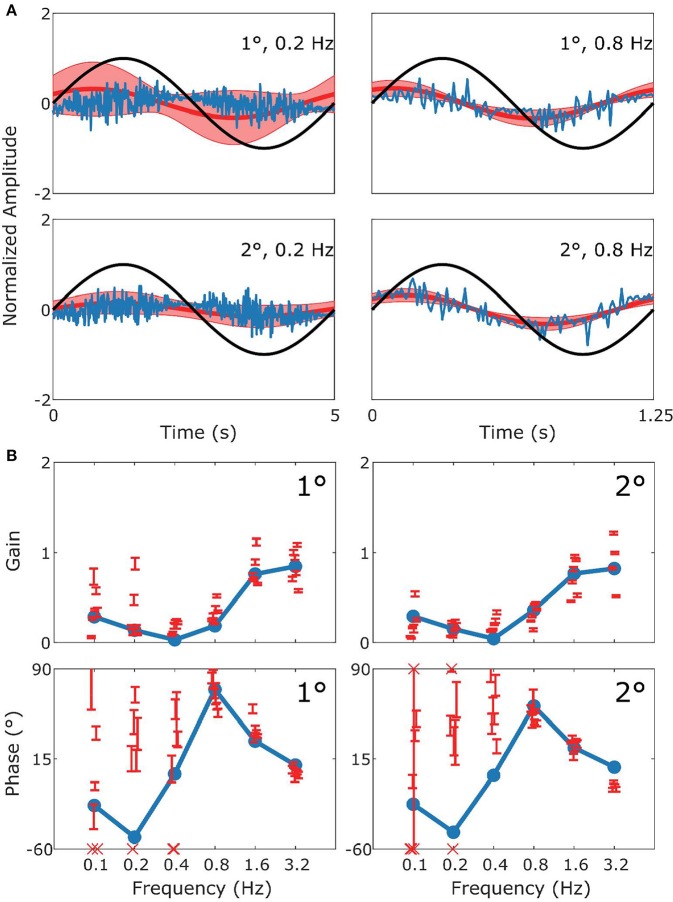
Summary of sVOR data and simulation. This figure follows the format of Figure 3, with panel **(A)** displaying the stimulus (black), model response (blue) and measured response (red). **(B)** Are Bode plots for Gain (top panels) and Phase (bottom panels). Note that the phase response of stimuli with Gains < 0.25 could often not reliably be determined. The pattern of the response in the behavioral data is clearly captured by the simulation.

The behavioral data that we present are in agreement with the values that have been previously published for the C57BL/6 mouse strain (Stahl et al., 2000; Faulstich et al., 2004; van Alphen et al., 2010; Schonewille et al., 2011). The VOR (Figure 3) in the dark responded to high frequency stimulation, and the OKR (Figure 4) was mainly active in response to low velocity stimuli (van Alphen et al., 2001). The vVOR (Figure 5) was more or less veridical over the whole stimulus range while suppression in the sVOR (Figure 6) paradigm mainly happened at low frequency/velocity conditions.

In Figure 3 we show a comparison of experimental and simulated VOR. Figure 3A displays examples of a single cycle of the model output for four examples of specific stimuli. We can see that the model response falls mainly within the red shaded region which represents variance of the population of mice responding to the same stimulus. The high frequency noise in the model response are due to the addition of motor and sensory noise. The high frequency noise is not seen in the mouse response as it is an estimate of mean mouse behavior. A comparable estimated mean model response is shown in Supplement Figure 2. Bode plots of mouse and model response across multiple frequencies and amplitudes is shown in Figure 3B. The figure demonstrates that model output falls within the region of typical mouse behavior across a range of frequencies of stimulation, both in terms of response gain and phase. We see that there is a good match between simulation and average experimental response over the whole stimulus range. First, a high gain at high frequencies and lower gain at low frequencies is clearly observable. Furthermore, we see a phase lead at low frequencies which diminishes with increasing stimulus frequency. Whilst the model fit well with the average of the population of mice tested, there is considerable variation between individual mice's responses to the various stimulations. In Figures 3–6B we present the confidence limits of estimates of each individual mouse and display any mice outside the limits of the plot as cross symbols on the limits of the y-axis.

Figure 4 follows the same format as Figure 3 but compares simulation to experimental results for the OKR response. The simulation nicely predicts the main features of the OKR response. The gain decreases and the phase lag increases with increasing stimulus velocity.

Figure 5 shows how well simulations predict experimental data for combined visual and vestibular stimulation (vVOR). In both the simulation and experimental data, we observe high gain and almost no phase lead or lag between response and stimulus. These results show that VOR and OKR have complementary results, which allows the combined system to produce excellent compensation of the retinal slip.

Figure 6 depicts how the model fits experimental data generated during sVOR—suppression of the VOR response with visual input. The response in high frequencies looks very similar to that in VOR because OKR is not responsive in high frequencies (see Figure 4), and hence cannot suppress vestibular triggered response. At low frequencies, there is a very small response, because VOR has low gain and is further suppressed by OKR. At these low frequencies, where the gain is low and variable, the model systematically misrepresents the phase of the eye movement.

**Figure 7:**
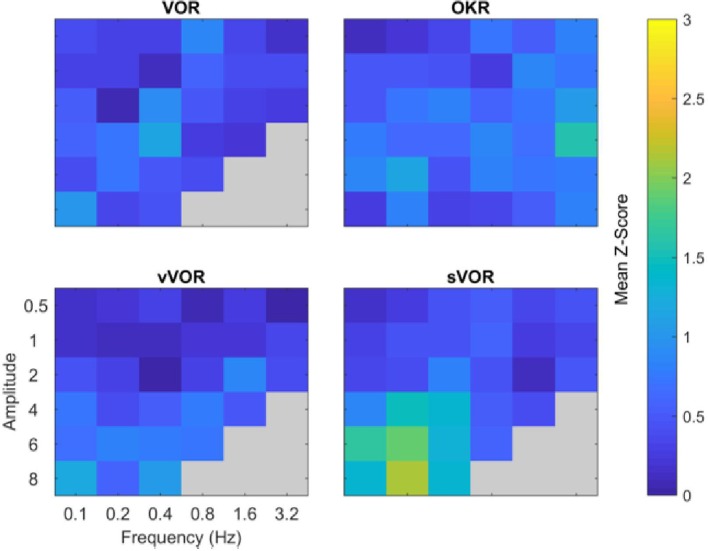
Summary of comparison of model and data for all amplitude and frequency combinations. The four panels depict the degree to which the model response matched the experimental data for the four conditions. The degree of similarity is expressed in terms of the number of standard deviations the model response was away from the mean behavioral response, cooler colors indicate a closer match. Across all amplitude and frequencies tested the model reproduces the experimental data well, with the possible exception of high amplitude, low frequency sVOR. Gray regions indicate conditions not measured in the experimental data.

In order to examine the overall quality of fit in each of the four experimental conditions above, we calculated the Z-scores of the overall fitting quality, these are displayed in Figure 7. Z-scores were calculated by subtracting the model response at each time point from the center of the region of typical mouse behavior and dividing by the standard deviation. Subsequently, we take the mean of these values across all timepoints. Therefore, the Z-score represents the number of standard deviations the model response is away from the mean mouse response. Note how the overall fit quality is good (“cool” colors in the heat map), with some poorer fits in the low frequency/high amplitude range of the sVOR condition. Because of the low amplitudes and high variability, the phase offset of the model at the lower frequencies does not lead to large Z-scores.

In addition to the comparison of model and data in terms of Z-scores (Figure 7), we also used the Bayesian estimates to generate probabilities for the model response falling outside the range of the behavior of a “typical mouse.” These tests were carried out for every frequency and amplitude combination and assessed the similarity of gain and phase separately, a combined probability was then generated from the product of these. The results of these tests are presented in the Supplementary Material. Overall the model responds within the range of a typical mouse for both gain and phase individually and when combined.

### Model Dynamics

**Figure 8:**
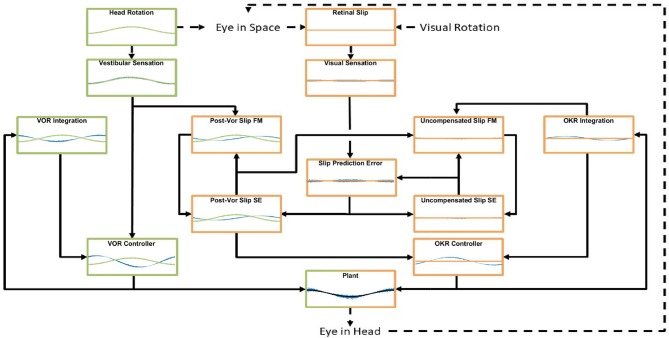
An example of the model dynamics for one cycle of the simulation in the vVOR condition (Stimulation amplitude of 2 degrees at a frequency of 0.2 Hz) at a time by which the system has reached a steady state. The layout matches the model schematic presented in Figure 1, Forward Models are labeled FM and State Estimators are labeled SE. In each box the blue line represents the output of the computation performed, the green or orange line represents the appropriate stimulus, vestibular and visual, respectively. In the supplement, we display the full model dynamics for the VOR and OKR conditions in isolation, respectively, for the same frequency and amplitude of stimulation.

The interaction of the different parts of the model in one of the conditions (vVOR, amplitude 2, frequency 0.2 Hz) are shown in Figure 8. The figure shows one cycle of the activity in each of the different areas being modeled during the steady state response to this stimulus. The top two boxes show that in this condition, the head is being rotated but the eyes are moving to keep the retinal slip at 0. The head rotation passes through the system in a feedforward manner to drive the vestibular controller. Additionally, this controller is modulated by knowledge of the eye position and velocity, driven by the forward model integration of the vestibular command. The figure also shows how the head rotation drives an estimate of the retinal slip that would remain uncompensated by the VOR controller. This is labeled post-VOR slip. Post-VOR slip in turn drives the activity of the OKR controller. Note that in this condition, the system estimates that the VOR will over-compensate for the head rotation and the OKR controller generator actually generates a command that is roughly in counter-phase with that of the VOR controller. The success of the vVOR in generating eye movements that fully compensate for the head movement are the result of a balance between the VOR signal and the OKR signal. Without the balancing OKR signal, the gain of the VOR would need to be lowered to achieve veridical tracking, which would compromise the quality of the VOR.

Figure 8 also shows that in this situation the OKR system has stabilized, such that retinal slip prediction error is 0. If there were prediction error, generated by either a transient visual or vestibular perturbation, this would drive an increase in the post-VOR slip which would then cause a transient increase in the OKR command to correct for the extra slip. The OKR system thus serves in two complementary roles: it generates a feedforward correction for the inaccuracies of the VOR system (the size of which is learned through adaptation, as described below) and it generates an error driven correction for unexpected retinal slip. The figure thus demonstrates the balance between the VOR command, post-VOR slip and OKR command that are necessary to achieve veridical tracking in the vVOR condition. Figures in the supplementary results show dynamic plots for the VOR and OKR simulations at the same frequency and amplitude, but it is their interaction which is the key innovation of our model.

### Sum of Sines

**Figure 9:**
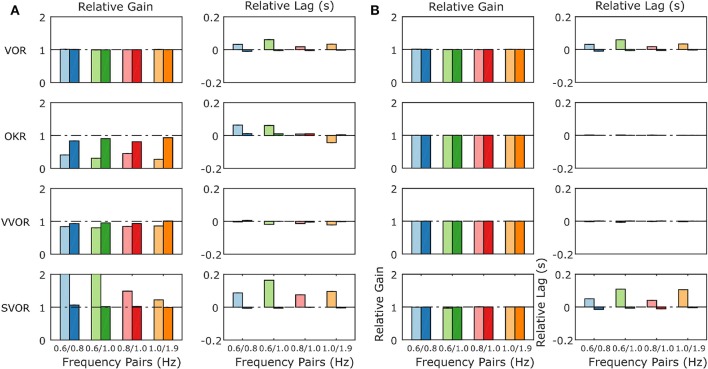
Summary of the model response to Sum of Sines stimulation for the model with normal retinal saturation **(A)** and with the saturation of retinal input removed **(B)**. The response is described in terms of gains and lags relative to the gain and lag recorded in response to the single frequency component presented in isolation. A linear system will produce only relative gains of 1 and relative delays of 0, indicated by dashed horizontal lines on each plot. The pattern of non-linearities produced by the full model **(A)** matches closely the non-linearities found in behavioral data in response to the same stimuli (Sibindi et al., 2016). Figure 6 of Sibindi et al. (2016) is reproduced with consent as a supplement to this figure, Supplement Figure 1. The removal of retinal saturation eliminates the non-linearities expressed in the relative gains of the OKR and sVOR but those expressed in the relative lags of VOR and sVOR remain intact. Please note that the values for relative gain for the 0.6 Hz component of the 0.6/0.8 and 0.6/1.0 Hz Sum of Sines in sVOR **(A)** are >2.

When the mouse OKR responds to sum-of-sines (SoS) stimuli, we have previously reported relative gain suppression of the lower of two frequencies in the stimulus. Conversely, in sVOR, results showed gain enhancement in the lower frequency component. In both sVOR and VOR, an overall decrease in phase lead was observed. For more details see Sibindi et al. (2016). When applying these stimuli to the model, the main pattern of effects is reproduced. Thus, we find qualitatively similar changes in both the relative gain and delay of the constituting frequencies (Figure 9A). Importantly, removal of retinal saturation (*R*_max_ = ∞) eliminates the non-linearities expressed in the gain of the response (Figure 9B).

### VOR Adaptation

Perhaps counterintuitively, VOR adaptation occurs as a result of changes in the OKR's model of VOR. Adaptation modifies the OKR's prediction of post-VOR slip. Thus, adaptation in our model involved allowing the parameter ζ to vary in response to retinal slip prediction error using gradient descent. As derived in the Supplementary Material, the gradient is in the direction that decorrelates head acceleration and retinal slip prediction error. The minimum error had a broad basin of attraction. Thus, regardless of the starting value of ζ, it always converged to the same value of −0.6, if the stimulation frequency was kept constant at 1 Hz. The value to which ζ converged depended on stimulus frequency but not amplitude. Nevertheless, for a broad range of frequencies ζ assumed a value around −0.6.

**Figure 10:**
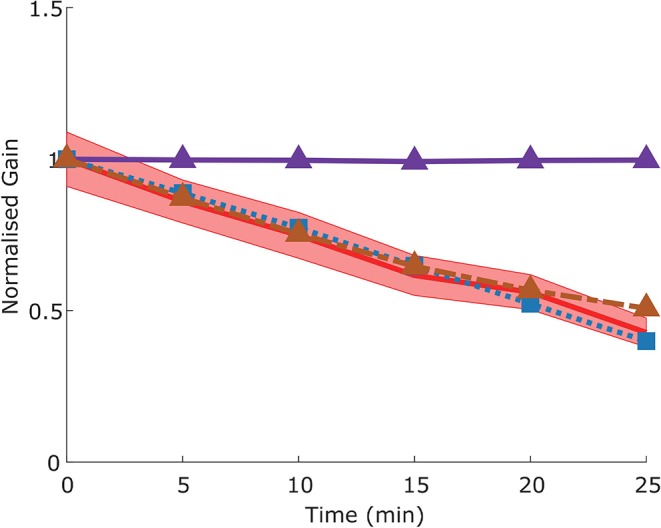
Time course of gain decrease adaptation of the VOR in response to repeated sVOR stimulation. The decrease in gain measured experimentally (red) with confidence limits representing SEM (shaded region) matches that produced by the model (blue line) in response to the same paradigm. Simulating a flocculus lesion in the model (purple line) by removing the four forward models produces a complete abolishment of adaptation, whereas an NPH lesion (orange line) left the adaptation intact.

The adaptation protocol reduced the gain of the VOR in mice to around 50% of its original value (Visible as a normalized gain of close to 0.5 after 25 min of training, Figure 10), comparable to that which has been previously described in literature (Schonewille et al., 2011).

### Effects of Lesions

In the model we simulated a lesion of the flocculus and a lesion of the NPH. The way in which this should be done in the model depends on the role that is ascribed to either structure (see section Discussion).

#### Flocculus Lesions

**Figure 11:**
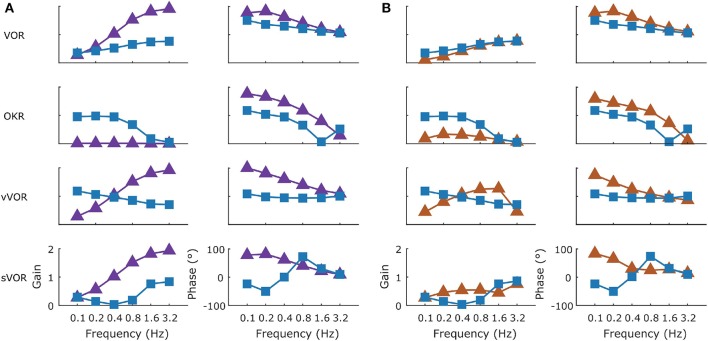
The effect of simulated lesions of the flocculus **(A)** and NPH **(B)** in the model on compensatory eye movements. The intact (blue line) and lesioned model response are summarized in Bode plots for the four conditions with the gain and phase presented in the left and right columns, respectively. Following a simulated flocculus lesion removal of the forward model stage produces an increase in the VOR gain and phase and an almost complete loss of the OKR response. Due to the loss of the OKR component the response in the vVOR and sVOR conditions is almost identical. Similarly, the greatest effect of a lesion of the NPH was on the OKR response with a large decrease in gain and decrease in phase lag.

We modeled a lesion of the flocculus by removing all the Forward Model boxes (Hexagon boxes in Figure 1). Figure 11A shows the result. The OKR is virtually absent. Meanwhile VOR gain is increased, and VOR phase increases at low frequencies. Following a model floccular lesion, the VOR did not adapt (Figure 10).

#### NPH Lesions

If one believes the NPH to be part of the controller (Green et al., 2007), a lesion of the NPH would mean removing the inputs of the two outer hexagonal Forward Model boxes of Figure 1. A lesion of the flocculus would then be setting the values of all Forward Model boxes to a constant value of 0.

Alternatively, if one believes the NPH is the oculomotor integrator (Cannon and Robinson, 1987), an NPH lesion means setting the output of [outer, hexagonal (Figure 1)] integration boxes to 0. A flocculus lesion then only affects the two inner FM boxes of Figure 1 (“post-VOR slip” and “uncompensated slip”). We tested both manipulations.

Both types of lesion of the NPH resulted in exactly the same result. This is not surprising, since they are equivalent to setting the input to the integration step to 0, or setting the output to 0. Both produced a small effect on the VOR with a decrease in gain at low frequencies, reflecting the mainly feed forward nature of response. OKR in contrast was greatly affected with a large decrease in gain (Figure 11B). As expected (see section Discussion), the lesion also had an effect on the drift of the eyes back to the center in the dark, decreasing the time constant from 2.83 to 0.31s. Stahl et al. (2006) report a time constant on the order of 5 s for the neural integrator in C57BL/6 mice, although there was considerable variation between mice and over time.

**Figure 12:**
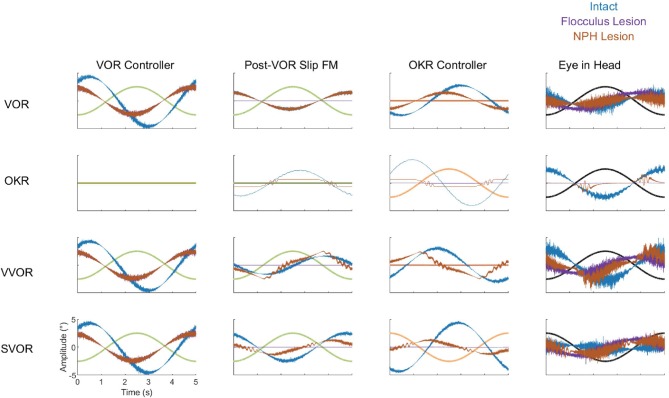
Summary of the dynamics of key components of the model in all four stimulation conditions (VOR, OKR, vVOR, and sVOR) for the full model (blue) and the simulated Flocculus (purple) and NPH lesions (orange). The first column of plots represents the output of the VOR controller, second column displays the forward model of Post-VOR Slip, the third and fourth columns depict the commands produced by the OKR controller and the Plant, respectively. In all plots the relevant stimulus is also displayed. The high frequency content of the control signals is due to the signal dependent noise in the sensory and motor stages of the model.

Cheron et al. (1986a,b) made lesions in the NPH of cats. They show that such a lesion reduces low frequency VOR responses and completely abolishes OKR. However, the gain and phase measurements do not depict the full nature of the changes in the response to OKR. When applying low velocity stimuli, the OKR in our model becomes noisy and dominated by oscillations at the time points in which stimulus velocity is highest (Figure 12).

In our model, NPH lesions do not affect adaptation to the sVOR stimulation at 1 Hz (Figure 10), because the individual reflexes at that frequency are relatively unaffected, and the site of plasticity is not lesioned.

#### Effect of Lesions on Dynamics

To better understand how the different lesions affect the internal dynamics of the model, Figure 12 presents the post-VOR slip and the activity of the visual, vestibular and combined controllers in each of the lesion conditions for each of the four different stimulus conditions. There are a number of key findings. First, both the floccular and NPH lesion have the same effect on the vestibular command. This is because both lesions impact the vestibular command by eliminating forward model estimation of eye eccentricity. This leads to a decreased amplitude and increased phase lag in the vestibular command. Meanwhile, it can be clearly seen that the NPH lesion primarily affects the magnitude of the OKR.

## Discussion

### Brief Summary of Results

Frens and Donchin (2009) proposed that CEM can be modeled by an SPFC framework where specific functional roles can be ascribed to specific nuclei in the CEM circuitry. Here, we measured—for the first time- VOR, OKR, vVOR, and sVOR over a large range of frequencies and amplitudes in the same animals. We then implement the SPFC framework in a detailed computational model which can, with a single set of parameters, mimic the behavior of OKR and VOR (Figures 3, 4, 7). With the same set of parameters, the model also reproduces vVOR, sVOR (Figures 5–7) and non-periodic SoS-stimuli (Figure 9). Furthermore, it successfully predicts the effects of lesions (Figures 11, 12) and has adaptive behavior, similar to VOR learning (Figure 10).

The strength of this model is that it has relatively few critical parameters (see Table 1) and that the critical parameters can be straightforwardly experimentally derived. This is an advantage over other SPFC-like models that address other motor systems (Shadmehr and Krakauer, 2008). However, it is important to recognize that although our model is in a tradition of modeling the motor system called optimal feedback models (Todorov, 2004; Shadmehr and Krakauer, 2008), this modeling approach does not assume that the motor system is actually optimal. Firstly, in biological motor control the correct cost function is unknown. Even if it were known, the biological motor system does not meet the criteria required for the optimization problem to be solved with available methods. Finally, optimal theories are difficult to falsify, as noted by the seminal paper of Shadmehr and Krakauer (2008). What the optimal control models in the neural control of movement share with true optimal feedback controllers is the basic structure of a feedback controller using forward modeling and a Kalman-like filter to produce state estimates that can be used to generate sensory prediction error. They also generally rely on an explicit or implicit cost function that balances control costs with target costs (Todorov, 2004). In the development of this model, we used a plausible cost function that could match the experimental behavior. We include both eye velocity and eye eccentricity. In combination with the signal dependent noise, the cost function penalizes larger movements. It is almost certainly not an accurate description of the real underlying cost function (see Harris and Waddington, 2013 for a more detailed approach at producing an accurate form), if indeed such a cost function exists. Our cost function follows Robinson (1981) in making the assumption that the system minimizes retinal slip. This assumption, reasonable in the afoveate, lateral eyed mouse, may not be appropriate for foveate species with saccadic systems or binocular vision. The expansion of the current model into other species is a worthy goal for future work but our aim in the current paper was to match the model to wealth of available data on the neuroanatomy and behavior for the mouse.

Another area in which we a priori limited our model was the investigation of only the steady state horizontal component of the CEM system. Other models have been developed to simulate the interaction of multi-dimensional stimuli (Laurens and Droulez, 2007; Laurens and Angelaki, 2017). The expansion of the current model to incorporate more degrees of freedom or translational stimuli is an important goal. The data presented here are all collected after the transient responses at the initialization of stimulation have abated. However, experimental data on the initialization and the termination of the CEM reflexes has previously been modeled (Raphan et al., 1979). Investigating if the current model can reproduce these effects is an important area for research. Despite these limitations, the range of stimuli we have tested and the publication of all data, model, and analysis code online means that we have provided a framework that people can work with to investigate more esoteric issues and expansions.

Key to the model are two distinct circuits for VOR and OKR. The VOR loop is relatively simple, and mainly consists of an integration step. In traditional models (for review see Glasauer, 2007), the OKR responds to actual retinal slip. However, due to the relatively long delay of the visual processing, the OKR response would then typically respond late. OKR state estimation in our model resolves this by predicting retinal slip. Both the VOR and the OKR loop contribute to this internal estimate of (uncompensated) retinal slip. This combined contribution is necessary, since the OKR assumes that the vestibular system will only partially resolve the retinal slip. While the reality may be more complex, the idea that the OKR models the VOR was the only way that we could explain the relatively high gains of both the OKR and VOR systems in isolation with the veridical gain of the two systems combined.

Finally, our model implements adaptation as a recalibration of this OKR estimate of VOR slip compensation. This helps explain why floccular lesions have a stronger direct effect on OKR but also disrupt VOR adaptation.

### The Non-linear Response to SoS Stimulation

In addition to reproducing the response to sinusoidal stimulation in a wide range of conditions, the model also matched responses to SoS-stimuli that are identical to those previously used by Sibindi et al. (2016). Strikingly, two non-linearities reported in the results of that study were reproduced: The first is that when confronted with a visual stimulus that consists of two non-harmonic sinusoids (e.g., the summation of 0.6 and 1.0 Hz sinusoids), the amplitude of the lower frequency is suppressed, independent of the absolute value of the constituent frequencies. This then also results in changes in the amplitudes in vVOR and sVOR conditions. The second is that the lag of the response to the lower frequency is larger, resulting in a delayed overall response. This can be seen for both VOR, OKR, and its combinations.

The model has one non-linearity specifically built in: the saturation of the visual motion sensitive neurons in the retina (see Equation 8 in the Supplementary Material, parameter *R*_max_). Explicitly removing this saturation eliminated the gain decrease and delay increase of the OKR and vVOR, but left the increased delays in the VOR and sVOR unaffected (Figure 9).

These modeling results support the hypothesis that Sibindi et al. used to explain their results: increased delays may be a result of the circuit properties. That is, they suggest the forward model fails to predict upcoming retinal slip in complex stimuli. Our results also support their hypothesis that the gain changes are probably the result of non-linear retinal processing.

### The Role of the Flocculus

The flocculus acts as a forward model for both the VOR and the OKR loop. However, the role it plays in each reflex is completely different. The flocculus is not critical for VOR performance, as animals lacking Purkinje cells do have an intact VOR although the amplitude of the response is significantly higher (van Alphen et al., 2001). While our model does include a forward model and state estimator for head velocity, this is only a formal result of the structure of the model. In fact, our model ignores the results of the forward model and uses the sensory information exclusively to determine head velocity. Thus, the role of the forward model (green hexagon in Figure 1) in this system is actually only to integrate eye velocity into eye position. For the OKR loop the forward model helps to overcome the delay in the OKR feedback loop, and it is crucial to provide information about the estimated post-VOR slip.

We mimicked lesioning the flocculus by removing the output of the forward models. This removed the capability of the system to predict upcoming retinal slip. As a result, the optokinetic response was virtually abolished whereas VOR gain substantially increased (Figure 11A). Lurcher-mice, a mutant strain that lacks Purkinje cells, have substantially lower OKR gains than their wild type littermates (van Alphen et al., 2002). Lurcher-mice results are also similar to a floccular lesion in our model in that VOR-gain is increased. Results on VOR gain in acute, non-genetic floccular lesions are mixed (Rambold et al., 2002).

We can understand the results showing increased VOR gain in Lurcher mice using our model: the OKR generally acts to suppress the VOR and a floccular lesion releases this suppression. This interpretation leads to the further prediction that floccular lesions will reduce the effect of visual suppression of the VOR, increasing gains in the sVOR. This is true in our model as well as being compatible with the literature (Takemori and Cohen, 1974; Zee et al., 1981; Belton and McCrea, 2000).

The change in phase of VOR response that is seen in Lurcher mice (van Alphen et al., 2002) can be modeled only if we include the VOR integration stage in the flocculus. This supports the view of Green et al. (2007) that the NPH provides an efference copy that is integrated in the flocculus (see below).

### The Role of the NPH

Our model provides a potential resolution to a debate about the role of the NPH in eye movement generation. In Robinson's inverse-model framework, the NPH is thought to act as the neural integrator for horizontal eye position. Such an integrator is necessary to provide the abducens nucleus with both velocity and position commands that are needed to overcome the low-pass filtering properties of the plant (Robinson, 1981). This view has been widely adopted by researchers in the oculomotor system. A critical finding supporting this view is from Cannon and Robinson (1987) showing that lesions of the NPH cause the eye to drift toward the center of the oculomotor range. This is compatible with the loss of an integrator that opposes the elastic restoring forces of the plant. However, more recently Green et al. (2007) showed that the burst tonic neurons of the NPH have activity that is nearly identical to that of the motor neurons in the abducens nucleus. Furthermore, these neurons have direct projections to the flocculus (Langer et al., 1985; McCrea and Baker, 1985; Belknap and McCrea, 1988). On the basis of these findings, they proposed that the NPH provides efference copy input to a cerebellar forward model (Green et al., 2007; Ghasia et al., 2008). This view was also incorporated in our SPFC (Frens and Donchin, 2009). Thus, in our model, an NPH lesion removes input to the forward models. However, when we lesion the NPH projection in our simulation (by removing efferent copy to the forward model or by removing its output), we found that we had reproduced the Cannon and Robinson (1987) result: the time constant of the drift was reduced. Hence, a lesion of the efference copy projection produces the same results as those thought to support the idea that NPH is an integrator. It seems that the Cannon and Robinson (1987) results are compatible with both models while recent anatomical and physiological findings support the idea of efferent copy.

### VOR Adaptation

Within our framework, VOR adaptation happens through adaptive changes in the forward model of VOR used by OKR. OKR assumes that VOR will correct a certain fraction of sensed head velocity. Determining the proportionality constant robustly led to the same value regardless of stimulus amplitude over a wide range of frequencies. When challenged with an adaptation stimulus, the model gradually changed its gain. Of course, the rate of adaptation could be set arbitrarily. Our setting led to an adaptation speed that is very similar to what we experimentally found in mice under identical experimental conditions. To our knowledge, we are the first to suggest that VOR adaptation reflects adaptation of a forward model of VOR output. However, the idea is compatible with the recent suggestion that VOR adaptation is driven by the motor consequence of retinal slip rather than the slip itself (Shin et al., 2014). Floccular lesions in our model abolish VOR adaptation, which is in line with the literature (Schonewille et al., 2010). NPH lesions do not affect adaptation at 1 Hz in our model, but to the best of our knowledge there is no literature to corroborate this finding.

Although our model is capable of adaptation, we believe that adaptation in the biological system is probably more complex than that in our model. Biological adaptation seems to reflect plasticity at multiple sites with multiple time constants (Porrill and Dean, 2007; Gao et al., 2012; Clopath et al., 2014). The introduction of more realistic adaptation and testing adaptation at higher and lower frequencies is an important future extension of the current model.

### Relationship to Other Models

The CEM system is a popular candidate for computational modeling due to the known anatomical substrates and the restricted degrees of freedom. Theories of motor control are primarily based on one of two main architectures. One theory suggests that the motor system relies on generating an ideal “desired movement” or “desired trajectory” that serves as a basis for subsequent control. Such an architecture faces a number of key challenges: generating the desired trajectory, translating it into motor commands, and correcting for deviations during online control. At the heart of such a system is an “inverse model” which translates desired movement into motor commands (Jordan and Rumelhart, 1992). For the CEM system, the desired movement is always the one which will fixate the gaze in space, minimizing retinal slip. The literature in the CEM system contains a long tradition of such models (for example: Robinson, 1981; Kawato and Gomi, 1992; Glasauer, 2007; Lisberger, 2009; Clopath et al., 2014). In general, a desired motor command is fed to the brainstem, which then acts as an “inverse plant,” i.e., it processes the command in order to overcome the low-pass properties of the extraocular muscles and tissues that are connected to the eye.

Our model shares the use of internal models and sensory prediction with the Merfeld Observer Model (Merfeld and Young, 1995; Karmali and Merfeld, 2012) and the models of Laurens and Angelaki (2017). However, we concern ourselves mainly with the interaction of the VOR and OKR. Raphan et al. (1979) have also modeled the interaction of these reflexes and our model shares many elements with theirs. However, we place the notion of internal models at the forefront of our approach. The key innovation in our model is the use of recurrent cerebellar-vestibular nuclei loops which enable the model to function correctly in the presence of considerable motor and sensory noise and in the presence of significant delays in sensory feedback. There exists anatomical evidence for such loops (Büttner-Ennever and Büttner, 1992) and proposals for their functional significance have been made previously (Porrill et al., 2004).

Since the optimal control framework was originally proposed as an approach to understanding vertebrate motor systems, models of this sort have been implemented in the control of various motor tasks. The implementations closest to our model are those that attempt to describe coordinated head and eye movements during gaze shifts (Todorov and Jordan, 2002; Sağlam et al., 2011, 2014). One somewhat similar model has been proposed to describe the CEM system (Haith and Vijayakumar, 2007). The Haith model is built largely to address adaptation to changing dynamics, an issue not addressed by our data or our model. Additionally, the Haith model is not confronted with actual data. In sum, our model is unique in a number of respects: (1) the extensive data with which it is challenged, including lesion data and non-sinusoidal data, (2) the idea that one of the main drivers of adaptation is compensation of the OKR system for predicted VOR error, (3) the development of a fully realized recurrent model of the CEM system in the spirit of the optimal control feedback framework.

## Data Availability Statement

The Matlab code and data required for replication of the analysis presented in this paper is available on the Open Science Framework website (https://osf.io/feq7c/).

## Ethics Statement

The animal study was reviewed and approved by Erasmus MC Animal Ethics Board.

## Author Contributions

PH, MG, and OD wrote the code of the model as well as the analysis routines. TS, KA, and SD recorded the mouse data under the supervision of PH, OD, and MF. PH, OD, and MF wrote the manuscript. MF and OD provided the grants that sponsored the project. All authors were involved in discussions about the data, the model, and the implications.

### Conflict of Interest

The authors declare that the research was conducted in the absence of any commercial or financial relationships that could be construed as a potential conflict of interest.
