# Supplementary material for: A Neuroanatomically Grounded Optimal Control Model of the Compensatory Eye Movement System in Mice
Source: Front Syst Neurosci. 2020 Mar 25;14:13. doi: 10.3389/fnsys.2020.00013 (PMC7109542; doi:10.3389/fnsys.2020.00013)
Supplement: Supplementary file 1 [file Data_Sheet_1.pdf]

Supplementary Material

Title: A neuroanatomically grounded optimal control model of the compensatory eye movement system in mice. Holland et al.

Overview of Model

This section describes the details of the model of the CEM described in the main text. The description provides all of the equations used in sufficient detail for the model to be implemented, although the actual Matlab code is available on the Open Science Framework website (https://osf.io/feq7c/). The model was implemented in Matlab R2016a (The MathWorks, Natick, MA). The time step for the simulation used was 1 ms.

This section is divided into subsections that describe the implementation of the plant and the control system. In the section on the plant, we describe both the effector and input implementations. The effector implementation is a model of how firing in the oculomotor nuclei affects muscle activation, and how that drives eye movement. The inputs we model are the vestibular and the retinal inputs to the system. The description of the control system is

divided into three parts: the actual state dynamics; the system's estimate of state; and the transformation of state estimate into motor command.

The plant

In this section we describe the dynamics of eye movement as a function of the firing rate of neurons in motor nuclei (OMN/AB) that project to eye muscles. Output of the OMN/AB innervates the horizontal rectus muscles, which are responsible for horizontal eye movements. These nuclei are reciprocally activated and project to muscles that move the eyes in opposite directions. Hence eye velocity depends on the difference between OMN and AB activities. The transfer function of these nuclei for the monkey has been described using the formula (Robinson, 1981):

$$T_p \dot{E} + E = Cu \tag{1}$$

(Where E is eye position, u is motor command from the OMN/AB, and C and T_p are the gain and time constants, respectively). The motor commands from the two nuclei were not separately modelled, but rather their activity was represented in a combined manner as the sum of two oppositely signed command signals.

Eq. (1) describes a leaky integrator with leakage time (in s). In monkey, T_p has been estimated at 0.24s and in rabbits it can be estimated from the work of Stahl and Simpson (1995) and more recently for mice in Stahl et al. (2015) to be 0.5s. We ran our simulation both with $T_p = 0.24$ s and with $T_p = 0.5$ s , and saw no difference in the results. For this paper, we present results using $T_p = 0.5$s (see Table 1). For the purpose of the model, we absorbed the constant C into the definition of u , so that our motor command was specified in °/s rather than in units of firing rate:

$$\dot{E} = u - \frac{1}{T_p} E \tag{2}$$

Sensory Signals

Compensatory eye movements are driven by two different sensory signals – vestibular and retinal. In this section we describe the biological processes behind these sensory signals and the numerical models that can be used to describe them.

Vestibular input

Vestibular input is created by the semicircular canals in the inner ear. We transformed the head velocity to sensory signal in three steps: linear filtering, velocity-sensitive transformation, and delay. At high frequencies, canals sense head rotation velocity with high accuracy. However due to the physical properties of the sensor, the accuracy is not good at low frequencies (Robinson, 1981). Thus, the semicircular canals can be best described as a high pass filter that acts on head velocity:

$$\dot{V}^{(1)} = -\frac{1}{T_v} V^{(1)} + \dot{H} \quad (3)$$

Where $V^{(1)}$ is the first stage of the neural signal generated by the velocity sensitive vestibular afferents (as opposed to V, the internal representation of head velocity) that are driven by the actual rotational head velocity, $\dot{H}$, and T_V is the filter constant that defines the effective sensitivity range of the afferents. The value of T_V differs between species. In mice this constant was measured in Yang and Hullar (2007). While they fit their data using a fairly complex transfer function (here reproduced in its original Laplace-domain notation):

$$0.09\frac{3.0s}{(3.0s+1)(0.007s+1)}(0.2s+1)^{0.03} \quad (4)$$

A first order approximation of the formula, and neglecting the leading constant, gives us Eq. (3). Over the relevant frequency range, the two functions are nearly identical, with $T_v = 3\sec$ for regular afferents of the horizontal semi-circular canal that project to the vestibular nucleus. Van Alphen et al. (2001) found that a lower time constant is needed to explain VOR experimental data. It is possible that additional filtering in the input synapses of the vestibular nucleus explains the difference between the constant measured in the afferents and that seen behaviorally. However. we found that our behavioral data was best matched by a constant very close to that found by Yang and Hullar (2007), $T_v = 4\sec$.

Subsequently, we introduced a delay and added noise:

$$\dot{V}_k^{+} = \dot{V}_{k-\delta_V}^{(1)} + n_{V,k} \quad (5)$$

The vestibular delay ($\delta_V = 2$ ms) represents the physical response time of the semi-circular canal and the neuronal transmission delay (Sohmer et al., 1999) . The noise ($n_{V,k}$) has a standard deviation proportional to the size of the vestibular signal (with constant of proportionality a_V , with the tilde, $\sim$,

meaning “distributes as” and $N(\mu,\sigma^2)$ is the normal distribution with mean μ and variance σ^2):

$$n_{V,k} \sim N\left(0,\ a_V^2 \dot{V}_k^2\right) \tag{6}$$

Since vestibular inputs depend only on head movement and head movement is determined by the experiment, the behavior of the system has no effect on vestibular inputs. Thus, we calculated these signals offline before running the simulations and introduced them directly as input.

Retinal Input

Visual information is provided by motion sensitive neurons in the retina (Yoshida et al., 2001). Those neurons sense local velocity of the image on the retina (often called retinal slip). In our experiments, the entire retina experiences the same retinal slip, and it is equal to:

$$R_k = \dot{H}_k + \dot{E}_k - \dot{T}_k \tag{7}$$

Where R is retinal slip velocity, in °/s, $\dot{T}$ the velocity of the visual surroundings in °/s, and $\dot{E}$ is the velocity of the eye relative to the head (generated as described above in Eq. (2).

The retinal motion sensitive neurons are linear in a limited range. In rabbits, sensitivity peaks at about 0.6 °/s (Oyster et al., 1972), with neuronal firing

rates increasing through this range, but then dropping off for higher velocities. At 10 °/s the neurons are unresponsive. Neurons in the AOS (the retinal target driving OKR) have shown similar properties (Soodak and Simpson, 1988) . Currently available data does not give the precise saturation point for the motion processing system of the mouse. In order to fit our data, our model assumes saturation of $R_{\max} = 0.65$ deg/sec and a piece-wise linear response function, representing a population code of neurons that individually drop off at values between 0 and R_{max}:

$$h\left(R_k\right) = \begin{cases} R_k & -R_{\max} \le R_k \le R_{\max} \\ R_{\max} & R_k \ge R_{\max} \\ -R_{\max} & R_k \le -R_{\max} \end{cases} \tag{8}$$

The processing of visual signals adds substantial delay to the retinal feedback (Collewijn, 1969). Our model uses the value of $\delta_R = 70$ ms proposed for the delay in mice (van Alphen et al., 2001) :

$$R^+_{k-\delta_R} = h\left(R_{k-\delta_R}\right) + n_{R,k} \tag{9}$$

With R^+_k the current internal representation of retinal slip, and $n_{R,k}$ being the retinal noise, which has standard deviation proportional to the retinal activation (with a constant of proportionality a_R^2):

$$n_{R,k} \sim N(0,\ a_R^2 R_k^2) \tag{10}$$

Full system dynamics

The above descriptions of the oculomotor plant and the retinal and vestibular input are combined to make a nearly linear state equation for the plant. Thus, we use a standard linear systems formulation (Frens and Donchin, 2009) with the state of the system at time k, x_k, undergoing a particular dynamics specified by the matrix A. In addition, the state is influenced by three factors: the command signal, u_k, affects the state through a matrix, B, that specifies how each part of the command signal influences each element of the state; the external input, z_k, represents the influence of the external world on the state; also, the state is influenced by noise, n_k. Finally, this state leads to sensory input (often called the observation), y_k, through a matrix, D. Altogether, this leads to what is called the system equations:

$$\begin{aligned} x_{k+1} &= Ax_k + Bu_k + z_k + n_k \\ y_k &= Dx_k \end{aligned} \tag{11}$$

These system equations are linear. Each piece of this equation is treated in detail in the paragraphs that follow.

The state at time step k is represented by the following vector:

$$x_k = \begin{bmatrix} H_k & \dot{H}_k & V_k & E_{V,k} & \dot{E}_{V,k} & T_k & \dot{T}_k & E_{R,k} & \dot{E}_{R,k} & R_k & R_{k-1} & \cdots & R_{k-70} & V_{k-1} & V_{k-2} \end{bmatrix} \quad (12)$$

The state includes time-delayed versions of the retinal and vestibular sensory signals. R_k represents the retinal input being generated at this instant (based on the current eye velocity) and R_{k-1} through R_{k-70} represent increasingly delayed versions. The observation matrix, Eq. (20), is such that only the fully delayed retinal slip, R_{k-70}, is available to the state estimation. The vestibular input is not affected by the behavior of the system, so it was generated offline according to Eq. (3) and delayed by 2 ms according to Eq. (5).

z_k is the external input and includes the change in the actual head velocity, vestibular sensory signal, and movement of the visual stimulus. These signals can all be generated offline before running the simulation. The vector can be written as:

$$z_k = \begin{bmatrix} 0 & \Delta\dot{H}_k & \Delta V_k & 0 & 0 & 0 & \Delta\dot{T}_k & 0 & 0 & 0 & 0 & 0 & \cdots & 0 & 0 & 0 & 0 \end{bmatrix}$$

(13)

n_k is the noise in the system. It affects eye velocity as well as vestibular and retinal input, so it can be written as:

$$n_k = \begin{bmatrix} 0 & 0 & n_V & 0 & n_u & 0 & 0 & 0 & n_u & n_R & 0 & 0 & \cdots & 0 & 0 & 0 & 0 \end{bmatrix} \quad (14)$$

In modelling the noise, we opted for model simplicity over realistic modelling of the noise. We followed the general idea in Todorov (2004) and Harris and Wolpert (1998) of having the size of the noise be proportional to the signal. Vestibular noise and retinal noise have already been described in Eqs. (6) and (10) respectively. The standard deviation of the motor noise is similarly proportional to the motor command (with constant of proportionality a_u)

$$\dot{E}_{k+1} = u_k - \frac{1}{T_p} E_k + n_u$$

$$n_u \sim N(0,\ a_u^2 u_k^2) \tag{15}$$

We ran the model with different constants of proportionality for the noise (a_u , a_R and a_V) up to 0.5 and did not see a change in the results. Given that we have no available data on amount of sensory or motor noise in the system we used values well in the middle of stable range, i.e.:

$$a_u = a_R = a_V = 0.1 \tag{16}$$

A is the matrix describing the state dynamics and is written as:

$$A=\begin{bmatrix}
1 & dt & 0 & 0 & 0 & 0 & 0 & 0 & 0 & 0 & 0 & 0 & \cdots & 0 & 0 & 0 & 0 \\
0 & 0 & 0 & 0 & 0 & 0 & 0 & 0 & 0 & 0 & 0 & 0 & \cdots & 0 & 0 & 0 & 0 \\
0 & 0 & 0 & 0 & 0 & 0 & 0 & 0 & 0 & 0 & 0 & 0 & \cdots & 0 & 0 & 0 & 0 \\
0 & 0 & 0 & 1 & dt & 0 & 0 & 0 & 0 & 0 & 0 & 0 & \cdots & 0 & 0 & 0 & 0 \\
0 & 0 & 0 & -\frac{1}{T_P} & 0 & 0 & 0 & 0 & 0 & 0 & 0 & 0 & \cdots & 0 & 0 & 0 & 0 \\
0 & 0 & 0 & 0 & 0 & 1 & dt & 0 & 0 & 0 & 0 & 0 & \cdots & 0 & 0 & 0 & 0 \\
0 & 0 & 0 & 0 & 0 & 0 & 0 & 0 & 0 & 0 & 0 & 0 & \cdots & 0 & 0 & 0 & 0 \\
0 & 0 & 0 & 0 & 0 & 0 & 0 & 1 & dt & 0 & 0 & 0 & \cdots & 0 & 0 & 0 & 0 \\
0 & 0 & 0 & 0 & 0 & 0 & 0 & -\frac{1}{T_P} & 0 & 0 & 0 & 0 & \cdots & 0 & 0 & 0 & 0 \\
0 & 1 & 0 & 0 & 1 & 0 & 1 & 0 & 1 & 0 & 0 & 0 & \cdots & 0 & 0 & 0 & 0 \\
0 & 0 & 0 & 0 & 0 & 0 & 0 & 0 & 0 & 1 & 0 & 0 & \cdots & 0 & 0 & 0 & 0 \\
0 & 0 & 0 & 0 & 0 & 0 & 0 & 0 & 0 & 0 & 1 & 0 & \cdots & 0 & 0 & 0 & 0 \\
\vdots & \vdots & \vdots & \vdots & \vdots & \vdots & \vdots & \vdots & \vdots & \vdots & \vdots & \vdots & \ddots & \vdots & \vdots & \vdots & \vdots \\
0 & 0 & 0 & 0 & 0 & 0 & 0 & 0 & 0 & 0 & 0 & 0 & \cdots & 0 & 0 & 0 & 0 \\
0 & 0 & 0 & 0 & 0 & 0 & 0 & 0 & 0 & 0 & 0 & 0 & \cdots & 1 & 0 & 0 & 0 \\
0 & 0 & 1 & 0 & 0 & 0 & 0 & 0 & 0 & 0 & 0 & 0 & \cdots & 0 & 0 & 0 & 0 \\
0 & 0 & 0 & 0 & 0 & 0 & 0 & 0 & 0 & 0 & 0 & 0 & & 0 & 0 & 1 & 0
\end{bmatrix} \tag{17}$$

Rows 2, 3 and 7 (velocity of the surroundings and of the head and the vestibular signal) are all just equal to 0. This reflects the fact that these variables are controlled by the inputs and not part of the dynamics of the system, in our model. Row 4 (eye position) simply includes the change in eye position caused by eye velocity (column 5), which needs to be scaled by $d = 0.001$ because eye velocity is in units of °/s and the time step is 1 millisecond. It is worth noting that row 8 also describes eye dynamics (just

like row 4). These representations are separated because in the internal controller they reflect different estimates. The simulation code keeps them in register by replacing them with the sum of the two values on each time step. Row 5 (and row 9) describe the tendency of the eye to drift back to center (the position dependent part of Eq. (2)). Row 10 says that current retinal slip is equal to head velocity plus eye velocity minus stimulus velocity (Eq. 7). The rest of the dynamics matrix (rows 13 through 78, not shown) simply shifts previous measured retinal input backwards in time

(e.g. $R_k \to R_{k-1}$, $R_{k-1} \to R_{k-2}$).

State transition is not, however, strictly linear. This non-linearity is represented by the function $h\left(Ax_k\right)$ in Eq. (8) so that,

$$h(Ax_{k+1}) = \left[H_k \quad \dot{H}_k \quad V_k \quad E_{V,k} \quad \dot{E}_{V,k} \quad T_k \quad \dot{T}_k \quad E_{R,k} \quad \dot{E}_{R,k} \quad h\left(R_k\right) \quad R_k \quad R_{k-1} \quad \cdots \quad R_{k-69} \quad R_{k-70} \quad V_{k-1} \quad V_{k-2}\right] \tag{18}$$

Where $h\left(R\right)$ describes the saturation of the retinal sensory signal (Eq. (8)). That is, every element of the state vector is preserved by h except the retinal slip which saturates.

Since the motor command, u_k, is a scalar, the control matrix B of Eq. (11) is a vector with the same size as the state. Because the command affects eye velocity directly, the only non-zero element of B is in the row representing

eye velocity. Units are adjusted so that 1 unit of motor command (neural activation) causes an acceleration of 1 °/ms, so B is:

$$B = \begin{bmatrix} 0 & 0 & 0 & 0 & 1 & 0 & 0 & 0 & 0 & 0 & \cdots & 0 & 0 \\ 0 & 0 & 0 & 0 & 0 & 0 & 0 & 0 & 1 & 0 & \cdots & 0 & 0 \end{bmatrix} \tag{19}$$

The second equation in Eq. (11) describes the observation, which is the part of the state available to the controller. The observation vector, y_k, contains delayed retinal and vestibular inputs. Thus, it can be calculated linearly using the observation matrix D (which is simply a 2x82 matrix of zeros with ones at locations (1, 82) and (2, 80) for vestibular and retinal input respectively). The D matrix is applied to the retinal slip after saturation, and we also add in sensory noise at this stage.

$$y_k = Dh(x_k) + \eta = \begin{bmatrix} \dot{V}_{k-\delta_V} + \eta_V \\ h(R_{k-\delta_R}) + \eta_R \end{bmatrix}^T = \begin{bmatrix} \dot{V}^+_{k-\delta_V} \\ R^+_{k-\delta_R} \end{bmatrix}^T \tag{20}$$

Control system

In this section we describe an optimal feedback controller for the compensatory eye movement system. This controller includes a forward model and a process of combining forward model prediction with sensory input, called state estimation. We will use the hat notation, $\hat{x}$, for estimates

produced by the forward model and the tilde notation, $\tilde{x}$, for the combined state estimate.

The operation of the controller can be described globally with the following equations:

$$\begin{aligned} \hat{x}_{k+1} &= A'\tilde{x}_k + Bu_k \\ \tilde{x}_{k+1} &= \hat{x}_{k+1} + K\left(y_k - h\left(D'\hat{x}_{k+1}\right)\right) \\ u_{k+1} &= -L\tilde{x}_{k+1} \end{aligned} \quad (21)$$

The first equation says that the forward model uses the previous state estimate and the previous motor command to generate a prediction of the next state. The second equation says that the estimate of the next state is generated by correcting this prediction for discrepancies between predicted and experienced retinal slip. The last equation says that motor command will be a linear function of the state. The tags on some symbols result from the fact that the controller's internal representation of state is different from the actual system state. Thus, A' is the internal representation of system dynamics and D' selects the appropriate sensory inputs from the internal system state.

VOR control

Our model assumes, as described in the main text, that VOR and OKR involve separate neural processing. Thus, it will be clearest if the operation

of each is described separately, and then the combined matrix equations will be easier to follow.

The architecture of the VOR is the same as the overall architecture of the system:

$$\begin{aligned}
\hat{x}_{\mathrm{V},k+1} &= A'_{\mathrm{V}}\tilde{x}_{\mathrm{V},k} + B_{\mathrm{V}}u_{\mathrm{V},k} \\
\tilde{x}_{\mathrm{V},k+1} &= \hat{x}_{\mathrm{V},k+1} + K_{\mathrm{V}}\left(\dot{V}_k^{+} - D'_{\mathrm{V}}\hat{x}_{V,k+1}\right) \\
u_{\mathrm{V},k+1} &= -L_{\mathrm{V}}\tilde{x}_{\mathrm{V},k+1}
\end{aligned} \tag{22}$$

In the case of VOR, since we have no access to the actual head velocity, we use the vestibular signal as an approximation of the head velocity. Thus, the state needs only have five elements:

$$\hat{x}_{V,k} = \begin{bmatrix} \hat{H}_k & \hat{\dot{H}}_k & \hat{\dot{V}}_k & \hat{E}_{V,k} & \hat{\dot{E}}_{V,k} \end{bmatrix} \tag{23}$$

The forward model is quite simple. The head velocity is not affected by either system dynamics or command (row 1 of Eq. 24 and the first 0 in Eq. 25). Eye movements have the usual plant dynamics (rows 4 and 5, which are taken from Eq. 2) and are affected directly by the motor command (the 1 in the third fifth position of Eq. 25):

$$A'_V = \begin{bmatrix} 1 & dt & 0 & 0 & 0 \\ 0 & 1 & 0 & 0 & 0 \\ 0 & 1 & 0 & 0 & 0 \\ 0 & 0 & 0 & 1 & dt \\ 0 & 0 & 0 & -1/T_P & 0 \end{bmatrix} \qquad (24)$$

$$B_V = \begin{bmatrix} 0 & 0 & 0 & 0 & 1 \end{bmatrix} \qquad (25)$$

The observation matrix returns the estimated head velocity (which is what we expect the vestibular input to be):

$$D'_V = \begin{bmatrix} 0 & 1 & 0 & 0 & 0 \end{bmatrix} \qquad (26)$$

This is compared to the actual vestibular input, $\dot{V}_k$. Because a floccular lesion does not eliminate VOR performance, we set $K_V = \begin{bmatrix} 0 & 1 & 1 & 0 & 0 \end{bmatrix}$. That is, the sensory feedback completely replaces the forward model in our knowledge of head velocity. The role of the forward model in this system is actually to integrate eye velocity into eye position.

Finally, the actual motor command is generated (see below for how these values are determined) using the equation $u_{V,k+1} = -L_V \cdot \tilde{x}_V$ with $L_V = \begin{bmatrix} 0 & 0.972 & 0 & -1.77 & 0.000233 \end{bmatrix}$ so that, ultimately:

$$u_{\mathrm{V},k+1} = -0.972\tilde{\dot{H}}_{\mathrm{V},k} + 1.77\tilde{E}_{\mathrm{V},k} - 0.000233\tilde{\dot{E}}_{\mathrm{V},k} \qquad (27)$$

OKR control

The job of the second part of the control loop is to estimate uncompensated retinal slip and compensate for it. Uncompensated visual slip arises from three sources: changes in the velocity of the visual stimulus, noise in the system, and head movements not compensated by the VOR. Importantly, the system cannot distinguish changes in the velocity of the visual stimulus from noise in the system. We use the symbol $\tilde{R}_k^*$ for the system's estimate of all three of these quantities together: the retinal slip uncorrected by VOR. We also call this the post-VOR slip, and it represents how much the visual environment would be moving in the absence of OKR.

The OKR's prediction of uncompensated retinal slip is thus the difference of two quantities: the post-VOR slip and the estimate of how much the OKR is moving the eye, $\hat{\dot{E}}_{R,k}$:

$$\hat{R}_k = \hat{\dot{E}}_{R,k} - \tilde{R}_k^* \tag{28}$$

The OKR system assumes that some amount of head movement will be compensated for by the VOR. Its estimate of uncompensated visual input generated by sensed head velocity is proportional to the actual sensed head velocity. Our forward model estimate of uncompensated post-VOR retinal

slip will be different from our previous estimate because it is updated by a factor proportional to head acceleration:

$$\hat{R}^*_{k+1} = \tilde{R}^*_k + \zeta\left(\hat{\dot{H}}_k - \hat{\dot{H}}_{k-1}\right) \tag{29}$$

(where ζ is the constant of proportionality and is discussed in the section on VOR adaptation below). We then use a Kalman filter to incorporate sensory prediction error and produce a final estimate of post-VOR retinal slip:

$$\tilde{R}^*_{k+1} = \hat{R}^*_{k+1} + K_{T,R_k}\left(R^+_{k-\delta_R} - \tilde{R}_k\right) \tag{30}$$

K_{T,R_k} represents the appropriate term in the Kalman gain matrix (specified fully below). Our data was best fit by using $\zeta = -0.6$ and $K_{T,R_k} = 0.05$ which means that that OKR has a tendency to overcompensate for head rotation and that it estimates that 5% of unexpected retinal slip represents real movement of the visual surroundings. Note that Eq. (30) also uses $R^+_{k-\delta_R}$ and $\tilde{R}_k$ which are the currently available retinal slip and its estimate while Eq. (28) and Eq (29) used $\hat{R}^*_k$ which is the estimate of the retinal slip happening right now. This estimate will be delayed for 70 ms before it becomes available as $\tilde{R}_k$. For the ease of the reader a supplement to Figure 1 (Figure S1-figure

supplement 1) includes a version of the model schematic with the various forms of retinal slip and its estimates labelled.

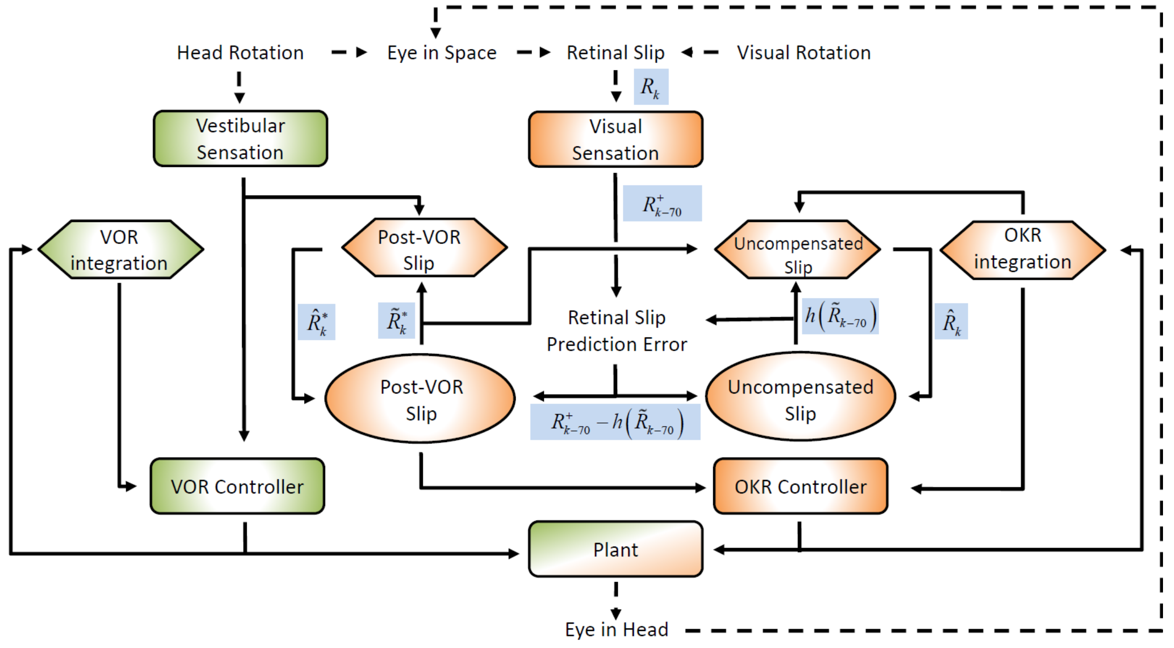

Figure 1-figure supplement 1. *Schematic representation of the model architecture with the different internal and external representation of retinal slip indicated in blue rectangles adjacent to the corresponding arrows. The color and shape coding of the figure is maintained from Figure 1 in the main text.*

With this understanding in place, we can describe the OKR control system. It has the same overall architecture as the full system:

$$
\begin{aligned}
\hat{x}_{R,k+1} &= A'_R \tilde{x}_{R,k} + B_R u_{R,k} \\
\tilde{x}_{R,k+1} &= \hat{x}_{R,k+1} + K_{T,R_k}\left(R^+_{k-\delta_R+1} - h\left(D'_R \hat{x}_{R,k+1}\right)\right) \\
u_{R,k+1} &= -L_R \tilde{x}_{R,k+1}
\end{aligned}
\tag{31}
$$

With function h representing the saturation of the retinal input (Eq. (8)). The state vector includes everything needed to calculate retinal slip, movement of the visual world, and head acceleration:

$$\hat{x}_{R,k} = \begin{bmatrix} \hat{H}_k & \hat{\dot{H}}_k & \hat{V}_k & \hat{T}_k & \hat{\dot{T}}_k & \hat{E}_{R,k} & \hat{\dot{E}}_{R,k} & \hat{R}_k^* & \hat{R}_k & \hat{R}_{k-1} & \cdots & \hat{R}_{k-69} & \hat{R}_{k-70} \end{bmatrix} \quad (32)$$

The forward dynamics matrix, A_R', look like this:

$$A'_R = \begin{bmatrix} 1 & dt & 0 & 0 & 0 & 0 & 0 & 0 & 0 & 0 & \cdots & 0 & 0 \\ 0 & 1 & 0 & 0 & 0 & 0 & 0 & 0 & 0 & 0 & \cdots & 0 & 0 \\ 0 & 1 & 0 & 0 & 0 & 0 & 0 & 0 & 0 & 0 & \cdots & 0 & 0 \\ 0 & 0 & 0 & 1 & dt & 0 & 0 & 0 & 0 & 0 & \cdots & 0 & 0 \\ 0 & \zeta & 0 & 0 & 0 & 0 & 0 & 0 & 0 & 0 & \cdots & 0 & 0 \\ 0 & 0 & 0 & 0 & 0 & 1 & dt & 0 & 0 & 0 & \cdots & 0 & 0 \\ 0 & 0 & 0 & 0 & 0 & -{}^{1}\!/_{T_P} & 0 & 0 & 0 & 0 & \cdots & 0 & 0 \\ 0 & \zeta & 0 & 0 & -1 & 0 & 0 & 1 & 0 & 0 & \cdots & 0 & 0 \\ 0 & 0 & 0 & 0 & 0 & 0 & 1 & 1 & 0 & 0 & \cdots & 0 & 0 \\ 0 & 0 & 0 & 0 & 0 & 0 & 0 & 0 & 1 & 0 & \cdots & 0 & 0 \\ \vdots & \vdots & \vdots & \vdots & \vdots & \vdots & \vdots & \vdots & \vdots & \vdots & \ddots & \vdots & \vdots \\ 0 & 0 & 0 & 0 & 0 & 0 & 0 & 0 & 0 & 0 & \cdots & 0 & 0 \\ 0 & 0 & 0 & 0 & 0 & 0 & 0 & 0 & 0 & 0 & \cdots & 1 & 0 \end{bmatrix} \quad (33)$$

These rows accomplish: calculation of uncompensated post-VOR retinal slip (row 8, implementing Eq. (29)), shifting of current vestibular input to previous vestibular input (rows 4-5), modelling of the eye plant (rows 6-7, implementing Eq. (2)), calculation of the current uncompensated retinal slip (row 9, implementing Eq. (28)). The rest of the A'_R matrix takes care of the delay of the estimated retinal slip.

The B_R matrix simply copies the motor command into the eye velocity vector, just as with the VOR system:

$$B_R = \begin{bmatrix} 0 & 0 & 0 & 0 & 0 & 0 & 1 & 0 & 0 & 0 & \cdots & 0 & 0 \end{bmatrix} \quad (34)$$

In calculating state estimation for the OKR system, we must take into account the non-linearity of the retinal processing before comparing the predicted retinal slip to the sensory input. We first use the matrix D'_{R} to select only the predicted uncompensated retinal slip, $\hat{R}_{k-69}$, from the state vector, as in Eq. (26) but with a larger state vector. Then, the predicted uncompensated retinal slip is cut off with the saturation function of the retinal input, as specified in Eq. (8). This can be compared to the true retinal input $R^{+}_{k-\delta R}$, providing retinal slip prediction error. The retinal slip prediction error updates the estimated state values of post-VOR retinal slip and uncompensated retinal slip. Our data was best fit by using:

$$K_R = \begin{bmatrix} 0 & 0 & 0 & 0 & 0 & 0 & 0 & 0 & 0.05 & 0.05 & \cdots & 0.05 & 0.05 \end{bmatrix} \tag{35}$$

Finally, the motor command is generated by using the equation $u_{R,k+1} = -L_R \cdot \tilde{x}_R$ just like in the case of VOR (again, see below for derivations), with $L_R = \begin{bmatrix} 0 & 0 & 0 & 0 & 0 & -1.77 & 0.000233 & 0.972 & 0 & 0 & \cdots & 0 & 0 \end{bmatrix}$ so that the motor command is:

$$u_{R,k+1} = -0.972\tilde{R}*_{R,k} + 1.77\tilde{E}_{R,k} - 0.000233\tilde{\dot{E}}_{R,k} \tag{36}$$

The combined controller: forward model

To produce a combined system, as described in Eqs. (21), in our calculations we simply combine the descriptions of the OKR and VOR systems above. The only state variable that overlaps in the two systems is the head velocity. However, this poses no difficulties.

$$\hat{x}_k = \left[\hat{H}_k \quad \hat{\dot{H}}_k \quad \hat{V}_k \quad \hat{E}_{V,k} \quad \hat{\dot{E}}_{V,k} \quad \hat{T}_k \quad \hat{\dot{T}}_k \quad \hat{E}_{R,k} \quad \hat{\dot{E}}_{R,k} \quad \hat{R}_k^* \quad \hat{R}_k \quad \hat{R}_{k-1} \quad \cdots \quad \hat{R}_{k-69} \quad \hat{R}_{k-70} \quad \hat{V}_{k-1} \quad \hat{V}_{k-2} \right] \tag{37}$$

And the dynamics and command matrixes can be copied from the two systems described above (the last sets of rows just shift the retinal slip and vestibular input back in time):

$$
A' = \begin{bmatrix}
1 & dt & 0 & 0 & 0 & 0 & 0 & 0 & 0 & 0 & 0 & 0 & \cdots & 0 & 0 & 0 & 0 \\
0 & 1 & 0 & 0 & 0 & 0 & 0 & 0 & 0 & 0 & 0 & 0 & \cdots & 0 & 0 & 0 & 0 \\
0 & 1 & 0 & 0 & 0 & 0 & 0 & 0 & 0 & 0 & 0 & 0 & \cdots & 0 & 0 & 0 & 0 \\
0 & 0 & 0 & 1 & dt & 0 & 0 & 0 & 0 & 0 & 0 & 0 & \cdots & 0 & 0 & 0 & 0 \\
0 & 0 & 0 & -\frac{1}{Tp} & 0 & 0 & 0 & 0 & 0 & 0 & 0 & 0 & \cdots & 0 & 0 & 0 & 0 \\
0 & 0 & 0 & 0 & 0 & 1 & dt & 0 & 0 & 0 & 0 & 0 & \cdots & 0 & 0 & 0 & 0 \\
0 & \zeta & 0 & 0 & 0 & 0 & 0 & 0 & 0 & 0 & 0 & 0 & \cdots & 0 & 0 & 0 & 0 \\
0 & 0 & 0 & 0 & 0 & 0 & 0 & 1 & dt & 0 & 0 & 0 & \cdots & 0 & 0 & 0 & 0 \\
0 & 0 & 0 & 0 & 0 & 0 & 0 & -\frac{1}{Tp} & 0 & 0 & 0 & 0 & \cdots & 0 & 0 & 0 & 0 \\
0 & \zeta & 0 & 0 & 0 & 0 & -1 & 0 & 0 & 1 & 0 & 0 & \cdots & 0 & 0 & 0 & 0 \\
0 & 0 & 0 & 0 & 0 & 0 & 0 & 0 & 1 & 1 & 0 & 0 & \cdots & 0 & 0 & 0 & 0 \\
0 & 0 & 0 & 0 & 0 & 0 & 0 & 0 & 0 & 0 & 1 & 0 & \cdots & 0 & 0 & 0 & 0 \\
\vdots & \vdots & \vdots & \vdots & \vdots & \vdots & \vdots & \vdots & \vdots & \vdots & \vdots & \vdots & \ddots & \vdots & \vdots & \vdots & \vdots \\
0 & 0 & 0 & 0 & 0 & 0 & 0 & 0 & 0 & 0 & 0 & 0 & \cdots & 0 & 0 & 0 & 0 \\
0 & 0 & 0 & 0 & 0 & 0 & 0 & 0 & 0 & 0 & 0 & 0 & \cdots & 1 & 0 & 0 & 0 \\
0 & 1 & 0 & 0 & 0 & 0 & 0 & 0 & 0 & 0 & 0 & 0 & \cdots & 0 & 0 & 0 & 0 \\
0 & 0 & 0 & 0 & 0 & 0 & 0 & 0 & 0 & 0 & 0 & 0 & \cdots & 0 & 0 & 1 & 0
\end{bmatrix} \tag{38}
$$

The internal representation of the command is two dimensional, with separate command for the VOR (dimension 1) and OKR (dimension 2), and each is added into the appropriate eye velocity:

$$
B' = \begin{bmatrix}
0 & 0 & 0 & 0 & 1 & 0 & 0 & 0 & 0 & 0 & 0 & 0 & & 0 & 0 & 0 & 0 \\
0 & 0 & 0 & 0 & 0 & 0 & 0 & 0 & 1 & 0 & 0 & 0 & & 0 & 0 & 0 & 0
\end{bmatrix} \tag{39}
$$

The combined controller: state estimation

In the second equation of the set in Eq. (21), the observation matrix, D', selects the vestibular and retinal input appropriately:

$$D' = \begin{bmatrix} 0 & 1 & 0 & 0 & 0 & 0 & 0 & 0 & 0 & 0 & 0 & 0 & \cdots & 0 & 0 & 0 & 0 \\ 0 & 0 & 0 & 0 & 0 & 0 & 0 & 0 & 0 & 0 & 0 & 0 & \cdots & 0 & 1 & 0 & 0 \end{bmatrix} \tag{40}$$

Note that the first row of D' is different than the first row of D. This difference comes from the fact that the internal system maintains an ongoing estimate of head velocity that is influenced by the input while the real system does not maintain such an ongoing estimate. The only representation of the delayed head velocity is the actual delayed head velocity. $h'(x)$ applies the retinal saturation non-linearity, $h(R)$ from Eq. (8), to the retinal slip and does not change the vestibular input:

$$h'\begin{pmatrix} \hat{\dot{H}} \\ \hat{R} \end{pmatrix} = \begin{pmatrix} \hat{\dot{H}} \\ h(\hat{R}) \end{pmatrix} \tag{41}$$

Parameters of the Kalman gain were selected by hand to match the data. We assumed that vestibular input only affects our estimate of the head velocity, $\tilde{\dot{H}}$, and that retinal input affects both our estimate of post-VOR retinal slip,

$\tilde{R}_k^*$, and our estimate of overall uncompensated retinal slip $\tilde{R}_k$ and its delayed versions. This gave the Kalman gain matrix the following form:

$$K = \begin{bmatrix} 0 & \kappa_V & \kappa_V & 0 & 0 & 0 & 0 & 0 & 0 & 0 & 0 & 0 & \cdots & 0 & 0 & 0 & 0 \\ 0 & 0 & 0 & 0 & 0 & 0 & 0 & 0 & 0 & \kappa_T & \kappa_{R,k} & \kappa_{R,k} & \cdots & \kappa_{R,k} & \kappa_{R,k} & 0 & 0 \end{bmatrix}$$

(42)

We set κ_V to 1, in order match the experimental finding that floccular lesion does not eliminate VOR. We set the other values to match the behavioral data. That is, the larger the value of κ_T and $\kappa_{R,k}$, the more quickly new retinal input affects our estimates. When the Kalman gains for the visual system are too large, noise reverberates in the system, leading to an explosion of noise in the OKR at low frequencies. When they are too low, the system does not manage visual following. Balancing these two considerations, we got the best match for our data with $\kappa_T = \kappa_{R,k} = \kappa_{R,69} = \kappa_{R,68} = \cdots = \kappa_{R,1} = \kappa_{R,0} = 0.05$.

The combined controller: cost function

We assumed that the primary goal of the optimal controller of the CEM in afoveate species (like rabbit and mouse) is to minimize motion of the visual field on the retina in order to stabilize the retinal image. We make the assumption that this cost is considered separately for VOR and OKR because

we are assuming that these reflexes are supported by separate neural substrates.

Thus, the overall cost of the system can be broken down into two parts, vestibular and retinal:

$$C = C_{\mathrm{V}} + C_{\mathrm{R}} \tag{43}$$

Each of the two sub costs is concerned with a different retinal slip: C_{V} relates to $\dot{\tilde{H}}_k + \dot{\tilde{E}}_{\mathrm{V},k}$, retinal slip due to uncompensated head motion, while C_{R} relates to $\tilde{R}_k^* + \dot{\tilde{E}}_{\mathrm{R},k}$, retinal slip due to uncompensated motion of the visual environment. In addition to the cost associated with retinal slip, each cost function includes a cost associated with eye eccentricities (this can be considered an "action" cost since eye eccentricity leads to extra muscle activity and energy expenditure). Finally, both cost functions discount future costs, as is common for an infinite horizon feedback controller: Thus, the two cost functions required for creating the two motor commands are:

$$\begin{aligned} C_{\mathrm{V}} &= \sum_{k=0}^{\infty} \gamma^{-k} \left(\left(\dot{E}_{\mathrm{V},k} + \dot{H}_k \right)^2 + \theta E_{\mathrm{V},k}^2 \right) \\ C_{\mathrm{R}} &= \sum_{k=0}^{\infty} \gamma^{-k} \left(\left(\dot{E}_{\mathrm{R},k} + R^*_k \right)^2 + \theta E_{\mathrm{R},k}^2 \right) \end{aligned} \tag{44}$$

The parameter θ balances between eccentricity and retinal slip costs. The parameter γ is the discount parameter Bradtke (1993) used to reduce the influence of increasingly distant costs. These two parameters were needed to match the drift of the eyes in the dark and were set to $\theta = 2$ and $\gamma = e^{\frac{1}{150}}$. For simplicity we approximated the infinite sum in Eq. (44) with a finite sum; we kept the first 100,000 terms.

The combined controller: the motor command

If our system had a linear plant (L), quadratic cost function (Q) and independent, identically distributed (i.i.d.) Gaussian noise, it would be called an LQR system (Åström and Murray, 2008). For such systems, it can be proven that the optimal controller can be separated in two independent parts – an observer and a simple controller – using the Ricatti equations (Lancaster and Rodman, 1995). We do not go into the details of these equations here, but we note that the CEM system, as described above, is not linear (because of non-linearities in the inputs) and does not have i.i.d. noise (since we use signal dependent noise). Nevertheless, the convenience of the LQR formulas has led to their frequent use in systems that are close to being LQR (Burns

and Ou, 1994; Lopez-Martinez et al., 2004). Previous experience is that this leads to nearly optimal controllers, and we followed this strategy here. However, before we apply Ricatti equations, we make one additional assumption. We assume that for the purposes of this solution, the controller assumes full correction of the head velocity by the VOR system. That is, we set $\zeta = 0$ in the matrix A', Eq. (38).

Applying the equations of Lancaster and Rodman (1995) to our system, Eq. (21), we derive a solution for the control policy, L.

$$L = \begin{bmatrix} 0 & 0.972 & 0 & -1.77 & 0.000233 & 0 & 0 & 0 & 0 & 0 \\ 0 & 0 & 0 & 0 & 0 & 0 & 0 & -1.77 & 0.000233 & 0.972 \end{bmatrix} \quad (45)$$

This can be more clearly written in terms of the final results for the motor commands:

$$u_{R,k+1} = -0.972\tilde{R}*_{R,k} + 1.77\tilde{E}_{R,k} - 0.000233\tilde{\dot{E}}_{R,k}$$

$$u_{\mathrm{V},k+1} = -0.972\tilde{\dot{H}}_{\mathrm{V},k} + 1.77\tilde{E}_{\mathrm{V},k} - 0.000233\tilde{\dot{E}}_{\mathrm{V},k} \quad (46)$$

The first term in both Eqs (46) compensates for retinal slip. The second term combines compensation for the "drift to center" generated by the elastic properties of the plant (Eq.(2)). This activity is apparently generated by the "neural integrator" produced by the firing of the tonic and burst-tonic premotor cells (Robinson, 1981). Experimental results presented in this

article and in other works (Cannon and Robinson, 1987) show the elastic properties of the plant are not fully compensated for by the controller; i.e. the neural integrator is leaky, and this leakage has a much higher time constant than the elastic term of the plant.

VOR adaptation

The parameter ζ (introduced in Eq.(29)) represents the extent to which the OKR system assumes head movements will go uncompensated. We model CEM adaptation as adaptation of this parameter so as to accurately predict retinal slip. The forward model prediction of retinal slip is given by Eq. (28). Where we recall that the star indicates that this is the estimate of the retinal slip that is we predict that is happening right now (post-VOR slip), as opposed to the estimate of the available retinal slip (with a 70 ms delay) which is indicated by $\hat{R}_k$.

We want to minimize the error in retinal slip prediction error (Figure 1-figure supplement 1):

$$Z_{R,k} = R^{+}_{k-70} - h(\tilde{R}_{k-70}) \tag{47}$$

We employ a decorrelation approach to adaptation (Porrill et al., 2013) and update ζ based on a factor proportional to the correlation of head acceleration and retinal slip prediction error.

$$\zeta_{\text{new}} = \zeta_{\text{old}} - \eta * Z_{R,k}\left(\hat{\dot{H}}_{k-1} - \hat{\dot{H}}_{k-2}\right) \tag{48}$$

Where η specifies the rate of adaptation. For the results presented here ζ was updated every 4 cycles of the stimulus (although this value is not critical and adaptation functions correctly with a wide range of update schedules) and was set to match the rate of adaptation in the experimental data: $\eta = 0.018$.

Experimental Methods

Animals

In order to test the model we recorded CEM in 13 C57Bl/6J mice (Charles River, Wilmington, MA, USA). All mice were housed on a 12h light / 12h dark cycle with unrestricted access to food and water. Experiments were performed during their light phase. All experiments were performed with approval of the local ethics committee and were in accordance with the European Communities Council Directive (86/609/EEC).

Surgery

Animals were prepared for head fixation by attaching two metal nuts to the skull using a construct made of a micro glass composite. The full procedure is described in van Alphen et al. (2009). Mice were given at least 3 days following surgery to recover before the start of any experimental paradigm.

Stimulus setup

Optokinetic stimuli were created using a modified Electrohome Marquee 9000 CRT projector (Christie Digital Systems, Cypress CA, USA) with a spatial resolution of at least 0.1 degrees and a temporal resolution of 0.01 s. The average luminance was kept constant at 17.5 cd/m^2. The stimuli were projected via mirrors onto three transparent anthracite-colored screens (156*125 cm), which were placed in a triangular formation around the recording setup (Fig 2A). This created a green monochrome panoramic stimulus fully surrounding the animal. The stimuli were programmed in C++ and rendered in openGL. They each consisted of 1592 green dots (2 degrees diameter) equally spaced on a virtual sphere with its center at eye height above the center of the table. Moving stimuli were generated by rotating the

virtual sphere around its vertical axis in sinusoidal patterns of different frequency and amplitude, so that all the dots moved coherently and in phase.

Vestibular stimulation was given by means of a motorized (Mavilor-DC motor 80, Mavilor Motors S.A., Barcelona, Spain) vestibular table that had its axis aligned with the center of the visual stimulus. The driving signal of both the visual and vestibular stimulation, which specified the required position, was computed and delivered by a CED Power1401 data acquisition interface (Cambridge Electronic Design, Cambridge, UK) with a resolution of 0.1 º and 0.01 s.

Eye movement recordings

Mice were immobilized by placing them in a plastic tube, with the head pedestal bolted to a restrainer that allowed translations in three dimensions such that the eye of the mouse was placed in the center of the visual stimulus and thus above the rotation axis of the turn table, in front of the eye position recording camera.

Eye movements were recorded with an infrared video system (Iscan ETL-200, Iscan, Burlington, MA, USA). Images of the eye were captured at 120

Hz with an infrared sensitive CCD camera [see van Alphen et al. (2009) for more details]. To keep the field of view as free from obstacles as possible, the camera and lens were mounted under the table surface, and recordings were made with a hot mirror that was transparent to visible light and reflective to infrared light (Fig. 2B). The eye was illuminated with two infrared LEDs at the base of the hot mirror. The camera, mirror and LEDs were all mounted on an arm that could rotate about the vertical axis over a range of 26.1^o (peak to peak). Eye movement recordings and calibration procedures were similar to those described by Stahl et al. (2000). Eye position was stored, along with the stimulus traces on hard disk for offline analysis.

Experimental Paradigms

Optokinetic Reflex

The OKR (N=9) was tested using visual stimuli, while the mouse was kept stationary. We presented sinusoidal stimuli containing a wide range of frequencies (0.1, 0.2, 0.4, 0.8, 1.6 and 3.2 Hz) and amplitudes (0.5, 1.0, 2.0, 4.0, 6.0 and 8.0^o), all about the earth vertical axis.

Vestibulo-ocular Reflex

The VOR (N=9) was tested with vestibular stimulation in the dark. Stimulus amplitudes and frequencies were identical to those used for the OKR, except that stimuli with a peak velocity higher than 60 °/s were discarded, because of mechanical considerations. Again, only rotations about the vertical axis were made.

Visually enhanced VOR and suppressed VOR

The vVOR (N=9) and the sVOR (N=6) protocols were identical to the VOR stimulation, except for the visual stimulation. During vVOR the visual stimulus was on, but stationary; during sVOR the visual stimulus was on and moved in phase and at the same amplitude as the turn table.

These four stimulus protocols were presented blockwise in 1 or 2 experimental sessions. Within each protocol the stimulus conditions were presented in random order to prevent effects of either learning or fatigue. All stimuli were presented for at least 5 cycles. The other protocols were performed separately.

Non-periodic stimulation

For non-periodic stimulation we opted to give Sum-of-Sine (SoS) stimuli. In these SoS conditions, the two constituent frequencies were chosen that had no harmonic relation. Four SoS frequency combinations were used in this study: 0.6/0.8 Hz, 0.6/1.0 Hz, 0.8/1.0 Hz and 1.0/1.9 Hz. Amplitude was either one or two degrees for each frequency component. Either both frequencies had the same amplitude (both 1º or both 2º) or they had different amplitude (one at 1º and the other at 2º). This led to a total of 24 types of stimuli in each of the OKR, VOR, vVOR and sVOR SoS conditions.

8 mice were used in this paradigm and they all performed all conditions.

Drift in the dark

In order to compute the plant time constant (see Supplementary Material, eq 15), we needed the mouse eye to drift in the dark from an eccentric position to the center of the oculomotor range. To do so, a visual scene moved slowly horizontal, thus making the eye move eccentrically. Subsequently, the light was turned off, and the mouse was in complete darkness. We then recorded the drift of the eye towards the center. By fitting an exponential function to this drift, the plant time constant was calculated. 6 mice were

measured over a range of drift amplitudes between 4 and 10 degrees, the number of drift repetitions was on average around 6 per amplitude per mouse.

VOR adaptation

VOR gain down adaptation (N=7) experiments consisted of 6 testing sessions and 5 training trials. Duration of each testing / training trial was 60s / 300s respectively. Sinusoidal (1 Hz, 5°) vestibular stimulation was applied in the dark for the testing sessions. During training sessions vestibular stimulation was accompanied by optokinetic sinusoidal stimulation of the same amplitude, phase and frequency (thus resulting in a stable head fixed visual surrounding).

Data Analysis

The Matlab (Matlab; The MathWorks, Natick, MA) code required for replication of the analysis presented in this paper is available on the Open Science Framework website (https://osf.io/feq7c/). Measured eye responses were analyzed offline. Position signals were transformed into velocity signals by a Savitski-Golay differentiating filter (cut-off frequency 50 Hz with a 3° polynomial) and were then smoothed with a median Gaussian filter (width 50

ms). Nystagmus fast phases and saccades were removed with a velocity threshold of 150°/s and with an FIR Butterworth low pass filter optimized to the stimulus frequency (cutoff at 3x stimulus frequency). There were two primary outcome measures in this study: gain and phase.

Gain and phase was extracted from the sinusoidal data by fitting a sinusoid and then using the gain and phase of the fit. The fit was done using a hierarchical Bayesian analysis using OpenBugs (Version 3.2.3, http://www.openbugs.net, [Lunn et al., 2009]). The precise details of the model used, as well as the parameters supplied to the OpenBugs algorithm, are provided below. In brief, the data for each trial for each mouse was assumed to be the result of a specific gain and phase specific to that trial, generated according to a distribution of gains and phases that were specific to the mouse. This distribution was, itself, generated according to hyper-parameters that characterize the population of mice. In addition, the noise in each trial was the result of a noise distribution characteristic of the mouse, which was generated according to hyper-parameters that characterized the population. Because our data was messy -- some mice had far more noise than others and some mice provided much more stable recording of eye movements than others -- the Bayesian approach allowed to incorporate all

of the data in a robust manner, discounting the noisy or incomplete data when making estimates of the population parameters. Ultimately, we show the 95% high density intervals for the gain and phase of the individual mice in the bode plots (Figures 3-6B).

In order to summarize the mouse population in Figures 3-6A we generated 10,000 samples of posterior predictive mice. That is, for each of the 10,000 Bayesian samples, we selected an amplitude and phase according to the parameters for the mouse population, and then used that amplitude and phase to generate sinusoidal data. We used these 10,000 ‘typical’ mouse sinusoids to define a region of typical behavior. We characterized this region using the mean and standard deviation of these movements at each time step.

To summarize the similarity of the model response and the mouse population as a single value for each stimulus condition we employed Z-scores. Using the typical behavior we then calculated a Z-score by subtracting the model response at each time point from the center of the region of typical behavior and dividing by the standard deviation. This Z-score was then averaged across time points for each condition.

For the non-periodic data, gain and phase information were obtained by fitting two sine waves to the stimuli and the data in custom-made Matlab curve fitting routines using the least squares method.

For all experiments the fits of the sine waves to the eye movement data provided the amplitude and phase of the eye movements. The gain was calculated as the ratio of the amplitude of eye movement compared to the amplitude of the stimulus, phase was calculated by subtracting the phase of the stimulus from the movement. Thus, a positive phase value indicates a leading eye position signal.

Statistics

Our statistics are geared to test whether the model behaves "similarly" to a typical mouse. This is different from the standard statistical test for effects and is also different from newly developed procedures that test for equivalence. We chose to test the confidence with which we could claim that model behavior lay within a "region of typical behavior" defined as the region within which 95% of mice are likely to fall. Thus, our p values represent the confidence with which we can make this statement.

For each condition, the gain and the phase of the model's behavior were compared to the posterior predictive distribution of gains and phases of the mice. That is, for each Bayesian sample, we took the population mean and the population standard deviation for the gain. This gave us, for each Bayesian sample, an estimate of the mouse typical parameter value, from the mean minus 1.96 times the population standard deviation to the mean plus 1.96 times the population standard deviation. We determined the percentage of samples for which the value of the gain in the model lay within this typical region. We used this as a measure of the posterior predictive probability that our model gain was similar to those of a typical mouse. We used an identical procedure for the phase.

Bayesian Fitting Procedure

The gains and phases of the single sine experimental data were estimated using a Bayesian fitting procedure using OpenBugs (version 3.2.3). The model used is specified in full form below:

```
model{
	for( rat in 1 : n.Rats ) {
		for( bin in 1 : n.Bins ) {
			for( rep in 1 : n.Reps ) {
```

```
                        Vel[rep , bin , rat] ~ dnorm(sint[bin , rat], tau.Vel.rat[rat])
                    }
                    sint[bin , rat] <- A.rat[rat] * sin(w * dT * bin - phi.rat[rat])
                }
                A.rat[rat] ~ dnorm(A.mu, A.tau)C(0,)
                phi.rat[rat] ~ dnorm(phi.mu, phi.tau)C(-π,π)
                tau.Vel.rat[rat] ~ dgamma(tau.Vel.shape, tau.Vel.scale)
        }
        A.mu ~ dunif(A.mu.lower, A.mu.upper)
        A.tau ~ dgamma(A.tau.shape, A.tau.scale)
        phi.mu ~ dnorm(phi.mu.mu, phi.mu.tau)C(-π,π)
        phi.tau ~ dgamma(phi.tau.shape, phi.tau.scale)
}
```

The fitting procedure was run with a burn-in of 500 samples, and then actual sampling of 10,000 samples in each of 3 chains. The initial values of the amplitude and phase of the fits were estimated from the data and each chain was initialized with a different precision (an order of magnitude between each). Convergence was assessed by manual inspection of the overlap of the chains and of the smoothness and overlap of the histograms for the posterior distribution of each parameter.

References

van Alphen, A.M., Stahl, J.S., and De Zeeuw, C.I. (2001). The dynamic characteristics of the mouse horizontal vestibulo-ocular and optokinetic response. Brain Res. *890*, 296–305.

van Alphen, B., Winkelman, B.H.J., and Frens, M.A. (2009). Age- and Sex-Related Differences in Contrast Sensitivity in C57Bl/6 Mice. Investig. Opthalmology Vis. Sci. *50*, 2451.

Åström, K.J., and Murray, R.M. (2008). Feedback Systems: An Introduction for Scientists and Engineers (Princeton, NJ: Princeton University Press).

Bradtke, S.J. (1993). Reinforcement Learning Applied to Linear Quadratic Regulation. In In Advances in Neural Information Processing Systems 5, (Morgan Kaufmann), pp. 295–302.

Burns, J.A., and Ou, Y.-R. (1994). Feedback control of the driven cavity problem using LQR designs. In , Proceedings of the 33rd IEEE Conference on Decision and Control, 1994, pp. 289–294 vol.1.

Cannon, S.C., and Robinson, D.A. (1987). Loss of the neural integrator of the oculomotor system from brain stem lesions in monkey. J. Neurophysiol. *57*, 1383–1409.

Collewijn, H. (1969). Optokinetic eye movements in the rabbit: Input-output relations. Vision Res. *9*, 117–132.

Frens, M.A., and Donchin, O. (2009). Forward models and state estimation in compensatory eye movements. Front. Cell. Neurosci. *3*, 13.

Harris, C.M., and Wolpert, D.M. (1998). Signal-dependent noise determines motor planning. Nature *394*, 780–784.

Lancaster, P., and Rodman, L. (1995). Algebraic Riccati Equations (Clarendon Press).

Lopez-Martinez, M., Diaz, J.M., Ortega, M.G., and Rubio, F.R. (2004). Control of a laboratory helicopter using switched 2-step feedback linearization. In American Control Conference, 2004. Proceedings of the 2004, pp. 4330–4335 vol.5.

Lunn, D., Spiegelhalter, D., Thomas, A., and Best, N. (2009). The BUGS project: Evolution, critique and future directions. Stat. Med. *28*, 3049–3067.

Oyster, C.W., Takahashi, E., and Collewijn, H. (1972). Direction-selective retinal ganglion cells and control of optokinetic nystagmus in the rabbit. Vision Res. *12*, 183–193.

Porrill, J., Dean, P., and Anderson, S.R. (2013). Adaptive filters and internal models: Multilevel description of cerebellar function. Neural Netw. *47*, 134–149.

Robinson, D.A. (1981). The Use of Control Systems Analysis in the Neurophysiology of Eye Movements. Annu. Rev. Neurosci. *4*, 463–503.

Sibindi, T.M., Holland, P.J., van der Geest, J.N., Donchin, O., and Frens, M.A. (2016). Superposition Violations in the Compensatory Eye Movement System. Investig. Opthalmology Vis. Sci. *57*, 3554.

Sohmer, H., Elidan, J., Plotnik, M., Freeman, S., Sockalingam, R., Berkowitz, Z., and Mager, M. (1999). Effect of noise on the vestibular system - Vestibular evoked potential studies in rats. Noise Health *2*, 41.

Soodak, R.E., and Simpson, J.I. (1988). The accessory optic system of rabbit. I. Basic visual response properties. J. Neurophysiol. *60*, 2037–2054.

Stahl, J.S., and Simpson, J.I. (1995). Dynamics of abducens nucleus neurons in the awake rabbit. J. Neurophysiol. *73*, 1383–1395.

Stahl, J.S., van Alphen, A.M., and De Zeeuw, C.I. (2000). A comparison of video and magnetic search coil recordings of mouse eye movements. J. Neurosci. Methods *99*, 101–110.

Stahl, J.S., Thumser, Z.C., May, P.J., Andrade, F.H., Anderson, S.R., and Dean, P. (2015). Mechanics of mouse ocular motor plant quantified by optogenetic techniques. J. Neurophysiol. *114*, 1455–1467.

Todorov, E. (2004). Optimality principles in sensorimotor control (review). Nat. Neurosci. *7*, 907–915.

Yang, A., and Hullar, T.E. (2007). Relationship of semicircular canal size to vestibular-nerve afferent sensitivity in mammals. J. Neurophysiol. *98*, 3197–3205.

Yoshida, K., Watanabe, D., Ishikane, H., Tachibana, M., Pastan, I., and Nakanishi, S. (2001). A Key Role of Starburst Amacrine Cells in Originating Retinal Directional Selectivity and Optokinetic Eye Movement. Neuron *30*, 771–780.

Supplementary Figures

Supplementary Figure 2

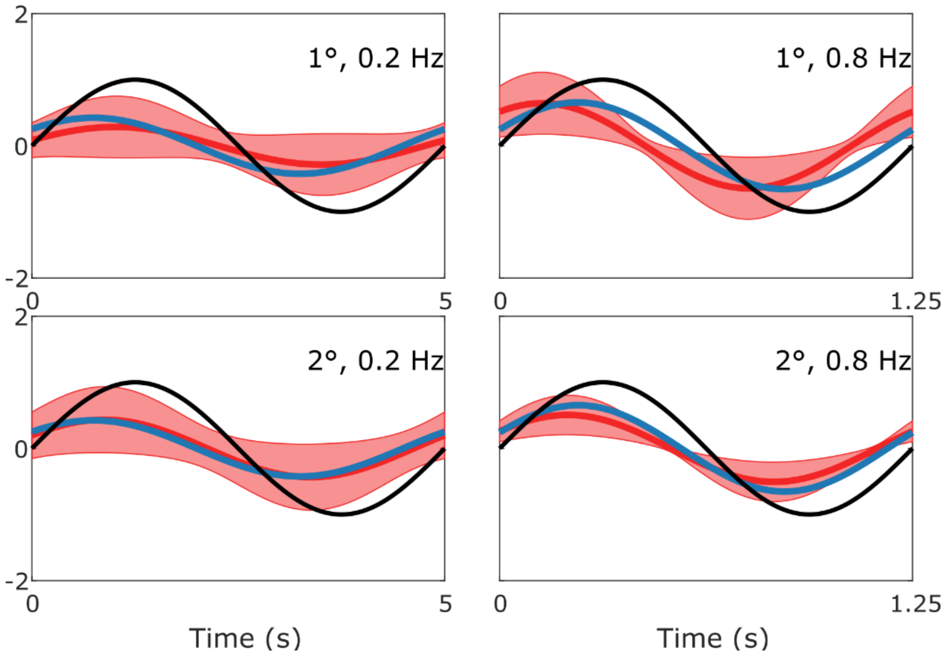

Figure 3-figure supplement 1. Response of the model (blue) and behavioural data (red) to a VOR stimulus (black). The format of the figure follows Figure3A, however, here the model response is displayed in the form of a sine wave fitted to the model output.

Supplementary Figure 3

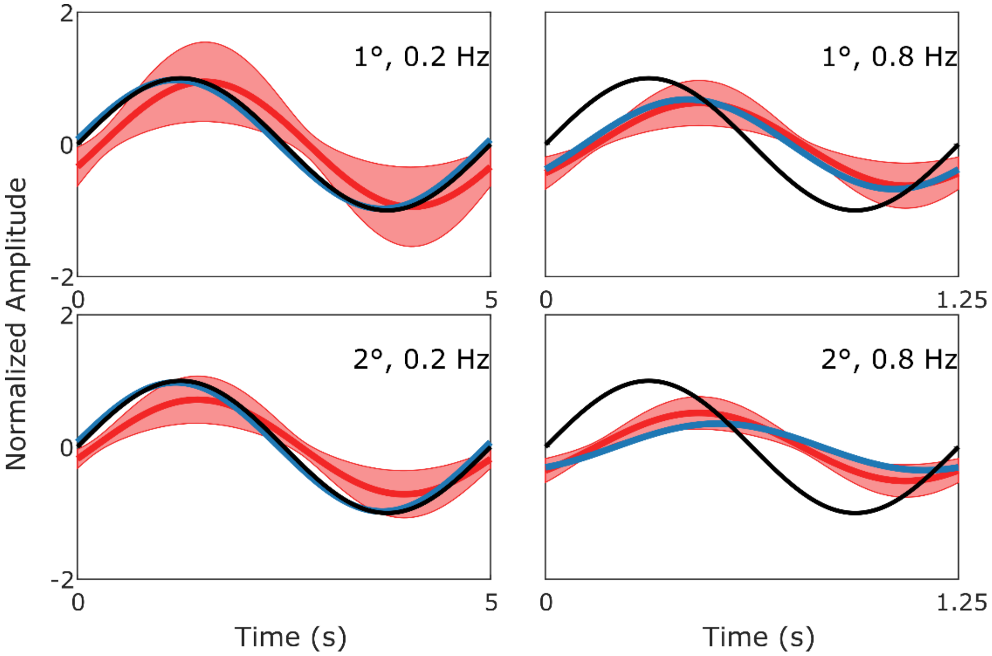

Figure 4-figure supplement 1. Response of the model (blue) and behavioural data (red) to an OKR stimulus (black). The format of the figure follows Figure4A, however, here the model response is displayed in the form of a sine wave fitted to the model output.

Supplementary Figure 4

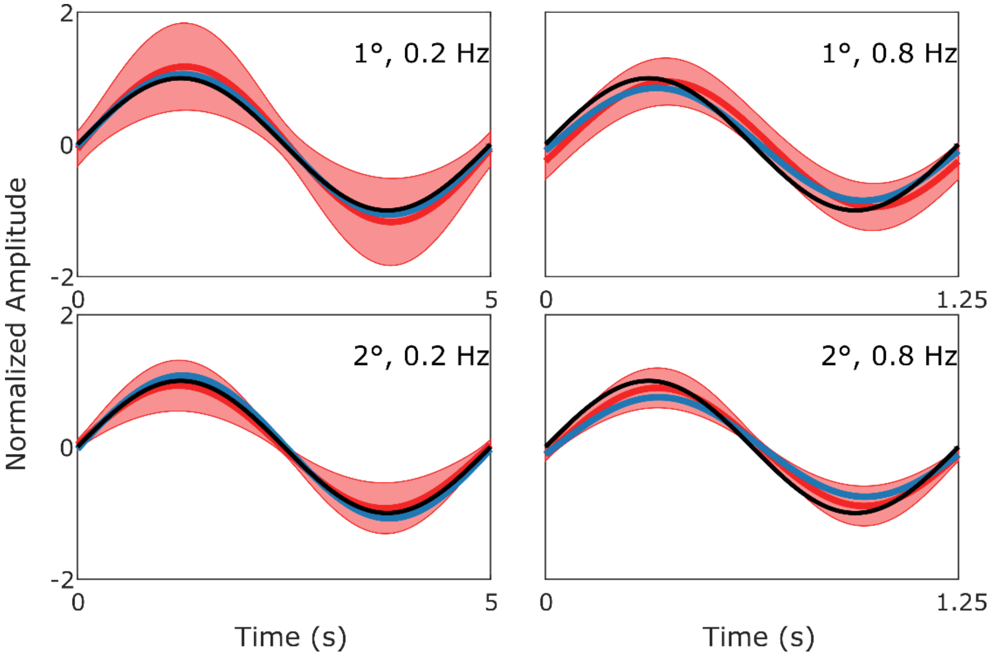

Figure 5-figure supplement 1. Response of the model (blue) and behavioural data (red) to a vVOR stimulus (black). The format of the figure follows Figure5A, however, here the model response is displayed in the form of a sine wave fitted to the model output.

Supplementary Figure 5

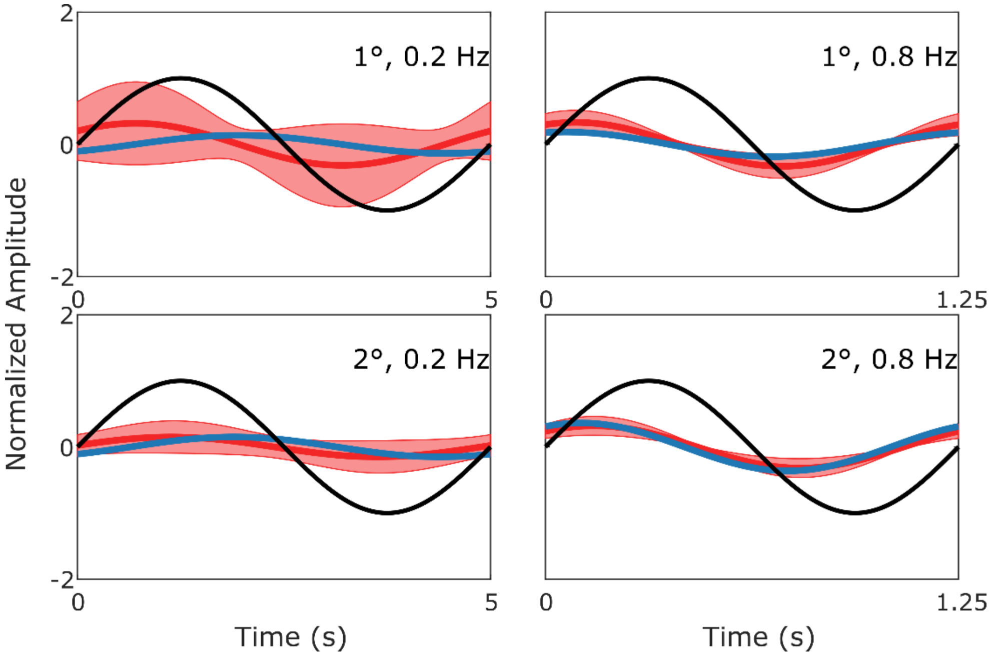

Figure 6-figure supplement 1. Response of the model (blue) and behavioural data (red) to a sVOR stimulus (black). The format of the figure follows Figure6A, however, here the model response is displayed in the form of a sine wave fitted to the model output.

Supplementary Figure 6

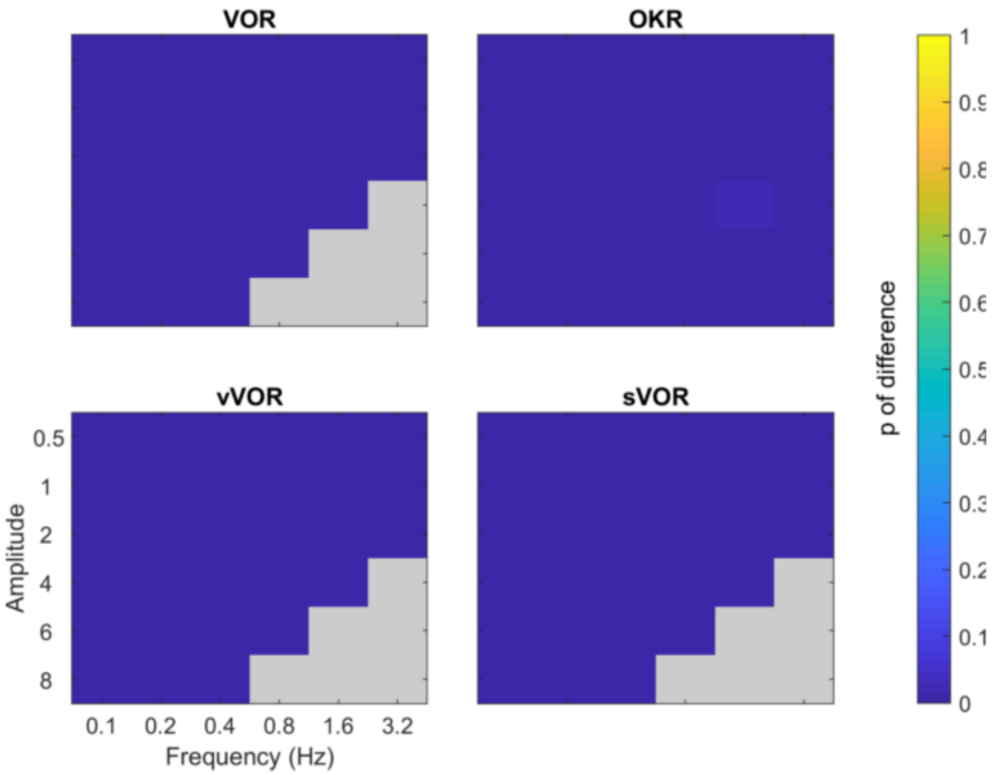

Figure 7-figure supplement 1. The combined probability that the model output falls outside the range of typical mouse behavior for every stimulus condition. The displayed probabilities represent the product of the probabilities of falling outside the range of typical behavior in gain and phase separately.

Supplementary Figure 7

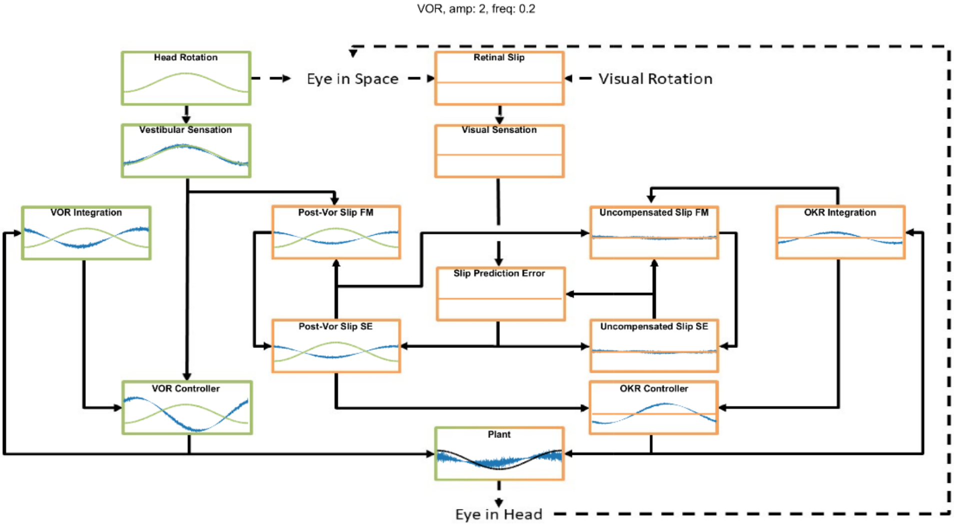

Figure 8-figure supplement 1. An example of the model dynamics for one cycle of the simulation in the VOR condition (Stimulation amplitude of 2 degrees at a frequency of 0.2Hz). The format of the figure matches Figure 8.

Supplementary Figure 8

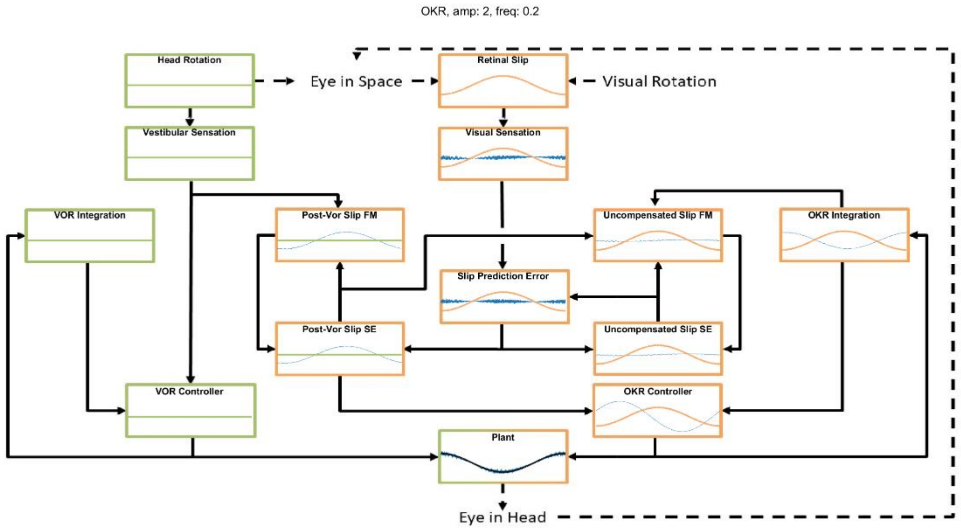

Figure 8-figure supplement 2. An example of the model dynamics for one cycle of the simulation in the OKR condition (Stimulation amplitude of 2 degrees at a frequency of 0.2Hz). The format of the figure matches Figure 8.

Supplementary Figure 9

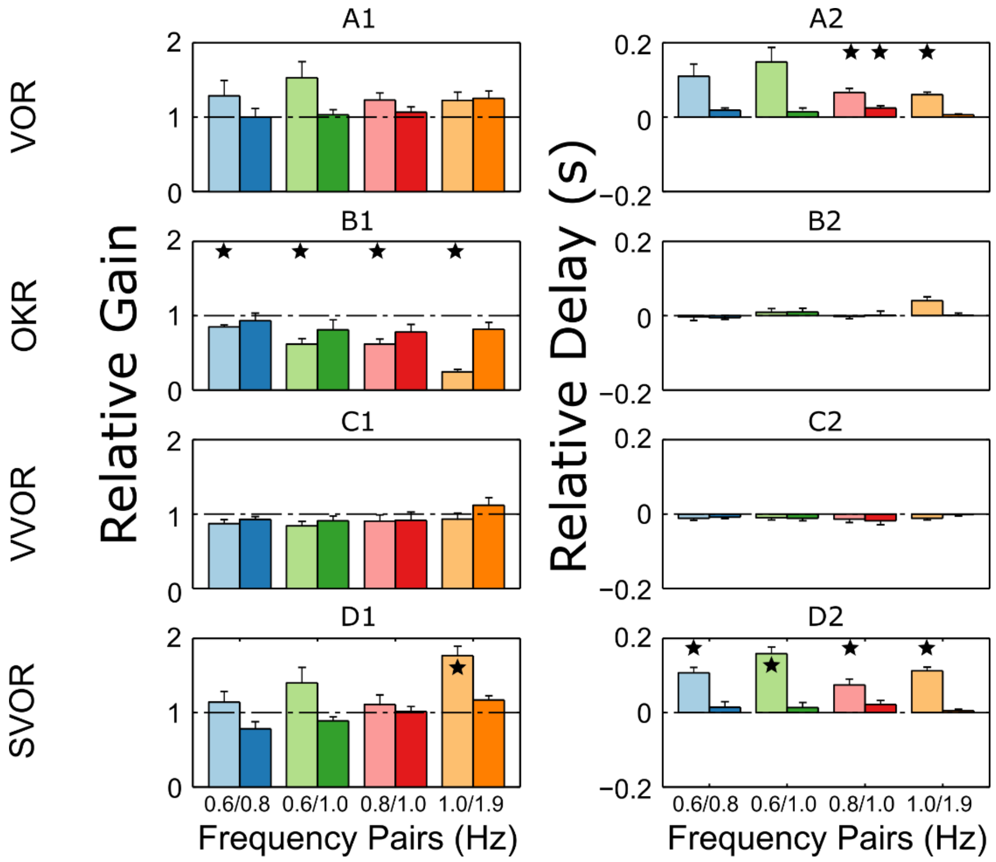

Figure 9-figure supplement 1. Behavioural response to Sum-of-Sines stimuli, reproduced with permission from Sibindi et al. 2016. The format of the figure follows Figure 9 and the stars on the bars indicate conditions in which non-linearity in the response was detected.
